# Macrophage uptake of oxidized and acetylated low-density lipoproteins and generation of reactive oxygen species are regulated by linear stiffness of the growth surface

**DOI:** 10.1371/journal.pone.0260756

**Published:** 2021-12-16

**Authors:** Erika J. Gruber, Ali Y. Aygun, Cynthia A. Leifer

**Affiliations:** Department of Microbiology and Immunology, College of Veterinary Medicine, Cornell University, Ithaca, New York, United States of America; Medizinische Universitat Graz, AUSTRIA

## Abstract

Macrophages are key players in the development of atherosclerosis: they scavenge lipid, transform into foam cells, and produce proinflammatory mediators. At the same time, the arterial wall undergoes profound changes in its mechanical properties. We recently showed that macrophage morphology and proinflammatory potential are regulated by the linear stiffness of the growth surface. Here we asked whether linear stiffness also regulates lipid uptake by macrophages. We cultured murine bone marrow-derived macrophages (BMMs) on polyacrylamide gels modeling stiffness of healthy (1kPa) and diseased (10-150kPa) blood vessels. In unprimed BMMs, increased linear stiffness increased uptake of oxidized (oxLDL) and acetylated (acLDL) low density lipoproteins and generation of reactive oxygen species, but did not alter phagocytosis of bacteria or silica particles. Macrophages adapted to stiff growth surfaces had increased mRNA and protein expression of two key lipoprotein receptors: CD36 and scavenger receptor b1. Regulation of the lipoprotein receptor, lectin-like receptor for ox-LDL, was more complex: mRNA expression decreased but surface protein expression increased with increased stiffness. Focal adhesion kinase was required for maximal uptake of oxLDL, but not of acLDL. Uptake of oxLDL and acLDL was independent of rho-associated coiled coil kinase. Through pharmacologic inhibition and genetic deletion, we found that transient receptor potential vanilloid 4 (TRPV4), a mechanosensitive ion channel, plays an inhibitory role in the uptake of acLDL, but not oxLDL. Together, these results implicate mechanical signaling in the uptake of acLDL and oxLDL, opening up the possibility of new pharmacologic targets to modulate lipid uptake by macrophages in vivo.

## Introduction

Atherosclerosis is a major underlying cause of cardiovascular diseases including stroke, coronary artery disease, and peripheral artery disease. Now widely understood as a chronic inflammatory disease, atherosclerosis develops due to progressive accumulation of cholesterol-rich low-density lipoproteins (LDL) and immune cells within the arterial wall that eventually form lipid-rich plaques [1]. The arterial wall undergoes extensive remodeling of the extracellular matrix (ECM) resulting in the characteristic global increase in arterial stiffness and focal regions of decreased stiffness [2–4].

Macrophages are the predominant immune cell type in atherosclerotic plaques, and scavenge infiltrating native low-density lipoprotein (LDL), as well as the oxidized (oxLDL) and acetylated (acLDL) forms that are cytotoxic and pro-inflammatory [5]. Uptake of oxLDL and acLDL occurs via multiple mechanisms, including phagocytosis and micropinocytosis; however receptor-mediated endocytosis is the dominant mechanism [6, 7]. Key receptors include scavenger receptor (SR)A, SRb1, CD36, and lectin-like oxidized LDL receptor-1 (LOX-1) [8–13]. Continued internalization of LDLs causes the formation of proinflammatory foam cells that secrete cytokines (e.g. tumor necrosis factor [TNF]α), generate reactive oxygen species (ROS), and eventually undergo necrosis to promote progression of disease [5, 14, 15]. Foam cells are central to plaque development in the ApoE^-/-^ mouse model of atherosclerosis [7, 16]. Because macrophages have a high degree of functional plasticity in response to microenvironmental cues, modulation of macrophage function has been proposed as a therapeutic mechanism to slow or reverse atherosclerosis [17, 18].

Arterial stiffness changes (measured by Young’s modulus of elasticity (pascals; Pa)) precede hypertension, and in patients with hypertension, atherosclerosis is associated with higher risk of death [19–21]. Regional differences in plaque occur in part due to ECM remodeling and result in stiffnesses that vary from soft lipid-rich areas (1–5 kPa) to stiff cellular fibrotic areas (10–14 kPa) to very stiff hypocellular fibrous cap regions up to 250kPa [3]. In a mouse model of atherosclerosis, increased arterial stiffness also precedes plaque development, and reduction of tissue stiffness reduces the accumulation of lipid-laden macrophages in the atherosclerotic plaque and subsequent plaque development [19, 22]. Global arterial stiffening increases vascular permeability and leukocyte transmigration and may increase the risk of cardiovascular disease [23].

In vitro growth substrates, such as polyacrylamide gels, can be adjusted to different stiffnesses to interrogate the role of stiffness in regulating cell function [24, 25]. We recently showed that macrophages on soft 1kPa polyacrylamide gels were more pro-inflammatory in response to toll-like receptor (TLR) activation compared to macrophages on gels of higher stiffness. Inhibition of rho-associated coiled coil kinase (ROCK1/2), a key mechanotransduction kinase that regulates diverse functions including cell migration, proliferation, and survival, enhanced TLR signaling and release of TNFα [26, 27]. Other groups have shown that increased linear stiffness correlates with enhanced lipopolysaccharide (LPS)-induced macrophage phagocytosis [28, 29]. Yet, the extent to which linear stiffness of the growth substrate regulates macrophage uptake of lipid is not well understood.

In this study, we evaluated the role of linear stiffness in regulating three major macrophage functions involved in the pathogenesis of atherosclerosis: phagocytosis, uptake of oxLDL and acLDL, and generation of ROS. We show that although phagocytosis by unprimed macrophage was unaffected by differences in linear stiffness, accumulation of both acLDL and oxLDL were profoundly affected. Stiffness-dependent increases in lipid accumulation correlated with increased surface expression of the lipoprotein uptake receptors CD36, SRb1, and LOX-1. Uptake of oxLDL was independent of ROCK1/2, but dependent on the major upstream signaling hub, focal adhesion kinase (FAK). In contrast, uptake of acLDL was independent of both ROCK1/2 and FAK. The mechanosensitive calcium ion channel, transient potential vanilloid-4 (TRPV4), which has been implicated in regulation of oxLDL uptake, negatively regulated the uptake of acLDL. Finally, we show ROS production is increased in both unstimulated and oxLDL-treated macrophages on stiff surfaces. Together, these findings identify mechanoregulatory mechanisms that differentially govern the uptake of acLDL and oxLDL.

## Materials and methods

### Reagents

Alexa Fluor 594-conjugated BioParticles^®^ synthesized from killed *Escherichia coli* (K-12 strain), *Staphylococcus aureus* (Wood strain without protein A), and Zymosan A (*Saccharomyces cerevisiae*) were from ThermoFisher Scientific (Waltham, MA). Silica beads were from Kisker Biotech (Moffat Beach, Queensland) and labeled with DQ Green BSA (ThermoScientific) as described [30]. Unlabeled oxLDL (lot number 910G18A), along with 1,1’-dioctadecyl-3,3,3’,3’-tetramethyl-indocarbocyanine perchlorate (DiI)-labeled acLDL (fl-acLDL; J65597; lot numbers 920C18A, 902F18A) and oxLDL (fl-oxLDL; J64164; lot numbers 920C18A, 902F18A) were from Alfa Aesar (Tewksbury, MA). Inhibitors of ROCK1/2 (Y-27632), FAK (PF-573228), and TRPV4 (HC-067047 and GSK2193874) were from Sigma-Aldrich (St. Louis, MO), and used at the indicated concentrations. Primary antibodies were against murine CD36 (monoclonal Armenian hamster; BioLegend; San Diego, CA), SRb1 (polyclonal rabbit; Novus biologicals; Littleton CO), and LOX-1 (polyclonal rabbit; abcam; Cambridge, MA). Dihydroethidium (DHE) was from ThermoFisher Scientific (Waltham, MA).

### Animals and cell culture

Murine (*Mus musculus*) primary bone marrow-derived macrophages (BMM) were generated from the femurs and tibias from 8–12 week old male wild type C57B/6 mice (WT; Jackson Laboratory, Bar Harbor, ME) or TRPV4 knockout C57B/6 mice (TRPV4-/-; originally generated by Suzuki et al. and kindly provided by Dr. David Zhang) [31, 32]. Bone marrow cells were plated at 0.8 x 10^6^ cells/ml in 10cm non-tissue culture-treated petri dishes in DMEM supplemented with 2 mM L-glutamine, 50 U/ml penicillin, 50 μg/ml streptomycin, 10% L cell conditioned media (v/v), and 10% low endotoxin FBS (v/v) (complete media) and cultured at 37°C in a humidified incubator with 5% CO_2_. BMMs were detached with 0.25% trypsin (Corning CellGro, Tewksbury, NY, USA), as needed. Cell viability was >95% (trypan blue exclusion) and cells were enumerated with a hemacytometer. Recovered cells were routinely >99% macrophages, as determined by F4/80 staining. All animal experiments were approved by Cornell University’s Institutional Animal Care and Use Committee (Animal Welfare Assurance A3347-01). Cornell University is accredited by the Association for Assessment and Accreditation of Laboratory Animal Care International.

### Preparation of polyacrylamide gels

Polyacrylamide gels of uniform thickness were prepared as described [26]. Briefly, polyacrylamide mixtures were prepared using stiffness-specific ratios of bisacrylamide:acrylamide in HEPES (14mM), and polymerized with tetramethylethylenediamine (0.0054%) and ammonium persulfate (0.05%) [25]. After polymerization, PA gels were coated with sulfa-SANPAH (sulfosuccinimidyl 6-(4’-azido-2’-nitrophenylamino)hexanoate) (0.2mg/mL, Sigma), and crosslinked with ultraviolet light for 10 minutes. Covalent binding of fibronectin (20μg/ml, Corning, Bedford, MA) to the gel surfaces was achieved with overnight incubation at 4°C. Lastly, PA gels were equilibrated in complete media at 37°C for 1 hour prior to cell attachment.

### Phagocytosis assay and microscopy

4 x 10^5 BMMs were transferred to fibronectin-coated PA gels (1kPa, 20kPa, 150kPa) or fibronectin-coated glass for 24 hours. Fluorescently labeled silica beads or microbial BioParticles^®^ were opsonized by incubating with mouse serum 30 minutes at 37°C on an orbital rotator. Beads and Bioparticles^®^ were washed in sterile phosphate buffered saline (PBS), quantified on a hemacytometer, and added to the macrophages at a ratio of 3:1 (beads) or 10:1 (Bioparticles^®^). The assay was synchronized with a two-minute centrifugation at 400 x *g* and cells were incubated at 37°C for the indicated times. Coverslips were rinsed in PBS, fixed in 3% paraformaldehyde (PFA) in PBS, permeabilized in 0.1% Triton-X, and blocked with 1% bovine serum albumin (BSA; Affymetrix) in PBS. BMMs were incubated with Alexa Fluor 488-phalloidin (165nM, ThermoFisher) in 1% BSA (w/v) in PBS for 20 minutes at 22°C to stain filamentous actin. Coverslips and PA gels were mounted onto glass slides with Prolong Anti-Fade with DAPI (ThermoFisher Scientific). Slides were imaged with an Axio Imager M1 microscope (Zeiss, Thornwood, USA) and an Axiocam MRm (Zeiss). A minimum of 50 phagocytic cells on each surface were analyzed; the number of particles per cell was quantified using Cell Counter plugin in ImageJ software (open source).

### RNA purification and quantitative real-time PCR (qPCR)

Total RNA was isolated and purified with TRIzol according to the manufacturer’s instructions (Thermo Fisher Scientific, Waltham, MA). RNA was quantified by spectrophotometry (Quawell Q3000, Palo Alto, CA). Residual genomic DNA was digested with DNase I (Invitrogen, Carlsbad, CA), and cDNA was synthesized using SuperScript III reverse transcriptase reagents (Invitrogen) according to the manufacturer’s instructions in a thermocycler (BioRad MyCycler, Hercules, CA).

Quantitative real-time PCR (qPCR) was performed in duplicate using Power SYBR Green master mix reagent (Applied Biosystems by Life Technologies) according to the manufacturer’s instructions on an Applied Biosystems 7500 Fast Real-Time PCR System (Life Technologies) in standard mode. Cycle parameters were: 1 cycle at 50°C for 2 min, 1 cycle at 95°C for 10 min, then 40 cycles at 95°C for 15 sec and 60°C for 1 min. Relative gene expression was calculated with the ΔΔCt method using 18S as the reference housekeeping gene. Primer sequences are listed in Table 1. Unless otherwise indicated, primers were designed with the Integrated DNA Technologies Realtime PCR Tool. All primers were purchased from Integrated DNA Technologies (San Diego, CA).

**Table 1:** Primer sequences for qPCR.

Gene	Forward	Reverse
**18S** [33]	5’GTAACCCGTTGAACCCCATT3’	5’CCATCCAATCGGTAGTAGCG3’
**LOX-1**	5’GCTATGGGAGAATGGAACTC3’	5’GCTCCGTCTTGAAGGTATG3’
**CD36** [34]	5’GATGACGTGGCAAAGAACAG3’	5’TCCTCGGGGTCCTGAGTTAT3’
**SRA**	5’CTCTCTACCTCCTTGTGTTTG3’	5’TCCATAGGACCTTGAGATGT3’
**SRB1**	5’AGTGGGGGTGGGAGAGAAAC3’	5’CAAGCCTGTGAGCCTGAAGC3’
**LDL-R**	5’GGCCATCTATGAGGACAAAG3’	5’TCAGCCACCAAATTCACAT3’
**ABCA1** [35]	5’ATAGTGTGGAGCTGCCCCATCA3’	5’CCACATCCTGCAAGTAGGCGAA3’
**ABCG1**	5’TGTTCTTCTCCATGCTGTTC3’	5’TCAGGCTGTACCAGTAGTT3’

### Flow cytometric analysis

BMMs were equilibrated to fibronectin-coated PA gels or fibronectin-coated glass. To measure lipid uptake, after 20 hours, media was replaced with OptiMEM supplemented with 1% BSA for 1 hour, and cells were then incubated with or without fl-acLDL (2μg/ml) or fl-oxLDL (2μg /ml) on the gels for 4 hours at 37°C. For inhibition studies, BMMs were pretreated with inhibitors (Y-27632, PF-573228, HC-067047, and GSK2193874) for 1 hour at 37°C prior to incubation with lipid. To measure surface protein expression of lipid uptake receptors, cells on PA gels for 24 hours were detached and nonspecific binding was blocked with unlabeled anti-CD16/CD32. Cells were incubated with unconjugated antibodies against CD36, SRb1, or LOX1 in PBS supplemented with 5% goat serum. After washing in 1% BSA in PBS, BMMs were incubated with goat anti-Armenian hamster Alexa Fluor 647(CD36) or goat anti-rabbit Alexa Fluor 488 (SRb1, LOX1) secondary antibodies. Negative controls were cells incubated with secondary antibody alone. To detect generation of reactive oxygen species (ROS), BMMs were equilibrated to gels and glass for 22 hours. They were then incubated with or without oxLDL (25mg/ml) in OptiMEM with 1% BSA for 2 or 24h at which time media was replaced with warm (37°C) PBS with or without dihydroethidium (DHE; 10μM) for 45 minutes. Cells were then washed and detached with 50mM EDTA in PBS. Single cell suspensions were prepared in Hanks’ buffered salt solution (HBSS, Corning, NY) with 1% BSA (w/v) and 0.1% sodium azide (w/v). Data were acquired with a BD FACSCantoTM II Flow Cytometer (BD Biosystems, San Jose), and a minimum of 10,000 events were analyzed using FlowJo software (FlowJo, LLC, Ashland, OR).

### Statistical analysis

Data were graphed and analyzed using GraphPad by Prism software (La Jolla, USA). Comparisons were made using unpaired t-test, Kruskal-Wallis, or one-way ANOVA with Tukey’s multiple comparisons test, as indicated in the figure legends. p < 0.05 was considered significant. Experimental replicate numbers are indicated in the figure legends.

## Results

### Phagocytosis in unprimed macrophages is not regulated by growth surface linear stiffness

**Fig 1:**
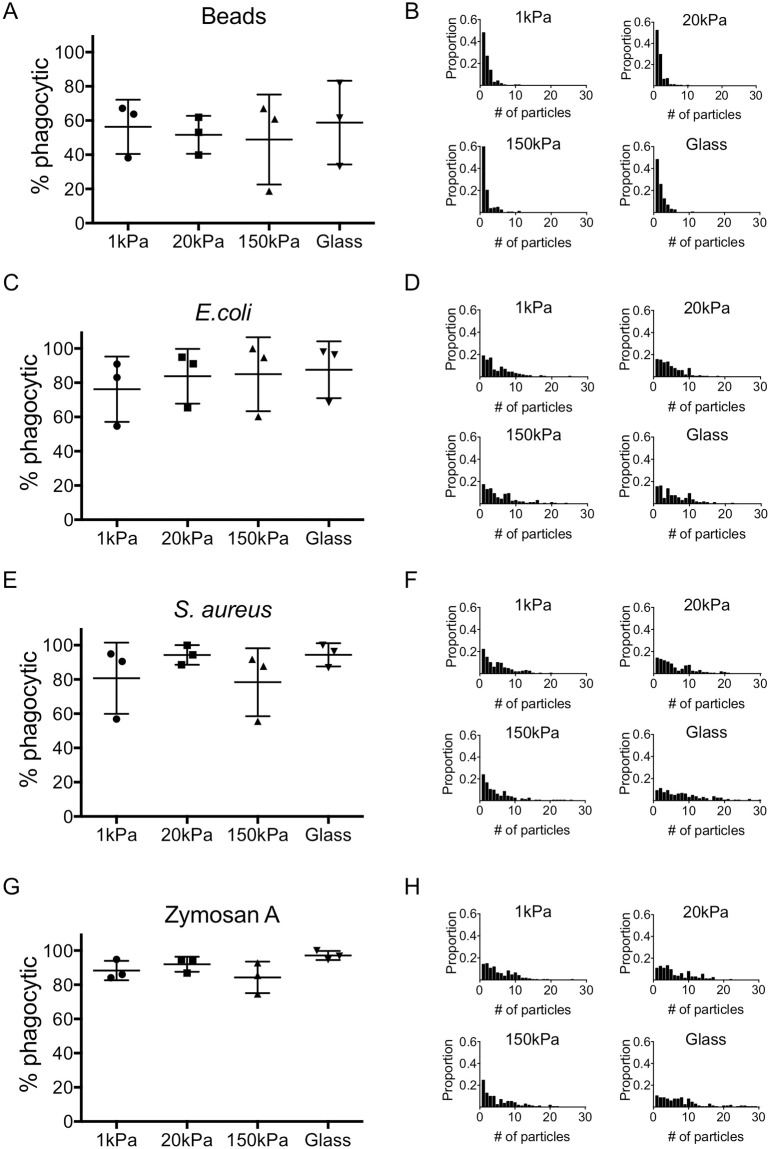
Phagocytosis in unprimed macrophages is independent of growth surface linear stiffness. Primary bone marrow-derived macrophages (BMMs) were grown on fibronectin-coated 1, 20, 150kilopascal (kPa) polyacrylamide gels or fibronectin-coated glass for 24 hours. BMMs were incubated for 30 min with fluorescently-labeled (A-B) silica beads, (C-D) *E*.*coli*, (E-F) *S*.*aureus*, or (G-H) Zymosan A particles for 30min, fixed in 3% paraformaldehyde, stained with phalloidin (F-actin), and imaged by epifluorescence microscopy. (A, C, E, G) show the percentage of BMMs on each of the surfaces that phagocytized at least one particle. Mean +/- standard deviation from three independent experiments is shown. Data were analyzed by one-way ANOVA with Tukey’s multiple comparisons test; no significant differences were reported. In (B, D, F, H), the number of internalized particles per cell from a minimum of 50 BMMs per condition in three independent experiments were quantified. Results were analyzed by the Kruskal-Wallis test.

Phagocytosis by plaque macrophages plays an important and complex role in the pathogenesis of atherosclerosis [36]. LPS-induced phagocytosis has been reported to depend on growth surface stiffness [28, 29], but it is unknown whether phagocytosis in the absence of LPS-priming is similarly regulated. Thus, we investigated phagocytosis of serum opsonized ~2μm silica beads and BioParticles (Gram-negative bacteria [*E*.*coli]*, Gram-positive bacteria [*S*. *aureus*], and Zymosan [*S*. *cerevisiae])* by BMMs equilibrated to fibronectin-coated 1, 20, 150kPa gels or fibronectin-coated glass. Fibronectin enrichment is one of the earliest changes to the ECM in atherosclerosis-prone regions [37, 38]. Around 60% of macrophages phagocytized at least one silica particle, and there was no difference in the percentage of phagocytic cells on any of the surfaces at either 5 minutes (S1A Fig) or 30 minutes (Fig 1A, representative microscopy images S2 Fig). Although the percentage of BMMs that phagocytized at least one particle appeared to be higher for each of the biologic particles compared to silica beads, this observation was only significant in BMMs on 20kPa PA gels incubated with *E*. *coli* (One-way ANOVA, *p*<0.05) and *S*. *aureus* (One-way ANOVA, *p*<0.01). For each of the biologic particles, we found no difference in the percentage of phagocytic cells on any of the surfaces at either 5 minutes (S1C, S1E and S1G Fig) or 30 minutes (Fig 1C, 1E and 1G).

On all surfaces, phagocytosis of a single silica bead was the most common observation (Fig 1B). BMMs on 20kPa gels phagocytized more beads than BMMs on 150kPa gels at 5 minutes; however, this difference was transient since there was no difference at 30 minutes (Fig 1B, and S1 Fig). At 5 minutes, the distribution of the number of *E*. *coli* particles per phagocytic BMM was slightly shifted to the right (more particles per cell) on 20kPa gels and glass compared to the other surfaces; this difference was gone by 30 minutes (Fig 1D and S1 Fig). These results differed from those observed with *S*. *aureus* or Zymosan A particles. No differences in the number of phagocytized of *S*. *aureus* or Zymosan A particles were detected at 5 minutes; however, at 30 minutes the distribution of the number of phagocytized *S*. *aureus* and Zymosan A BioParticles per BMM was shifted to the right on glass versus 1kPa, 20kPa, or 150kPa gels (Fig 1F and 1H, and S1 Fig). Although there were subtle differences in the number of particles phagocytized by BMMs on glass compared to gels, we conclude that, in general, phagocytosis in unprimed BMMs is independent of surface stiffness.

### Uptake of acetylated and oxidized LDL by macrophages is regulated by the linear stiffness of the growth surface

**Fig 2:**
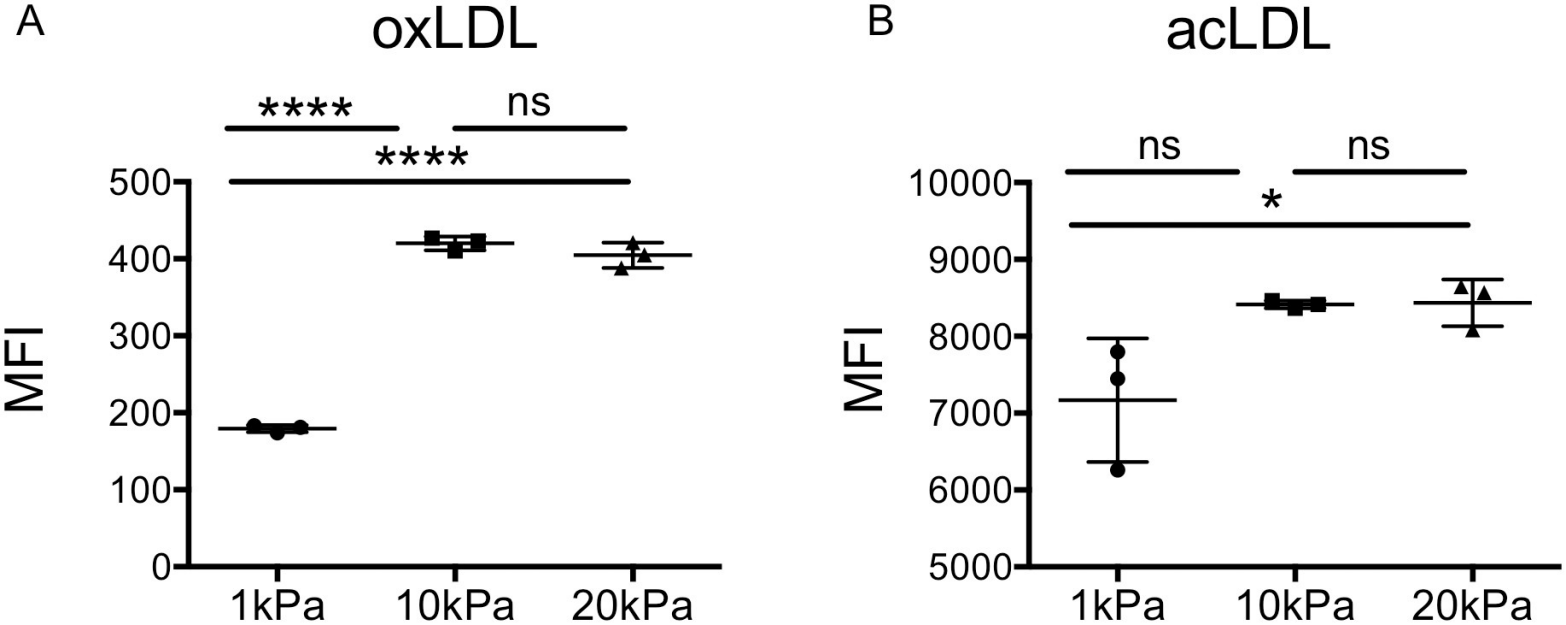
Uptake of oxidized LDL (oxLDL) and acetylated LDL (acLDL) is regulated by growth surface stiffness. Primary bone marrow-derived macrophages (BMMs) were grown on fibronectin-coated 1, 10, 20kilo pascal (kPa) polyacrylamide gels 24 hours prior to incubation with fluorescently labeled (A) oxLDL (fl-oxLDL, 2μg/ml) or (B) acLDL (fl-acLDL, 2μg/ml) for 4 hours. BMMs were removed into a single cell suspension for analysis of lipid uptake by flow cytometry. Graphs are representative of a minimum of three independent experiments. Mean +/- standard deviation of 3 biological replicates is shown. Data were analyzed with one-way ANOVA with Tukey’s multiple comparisons test. * p = 0.05; **** p < 0.001.

BMM accumulation of oxLDL (fl-oxLDL) and acLDL (fl-acLDL) is time- and dose-dependent (S3 Fig). We next asked whether the linear stiffness of the growth surface regulates macrophage uptake of fl-oxLDL or fl-acLDL. In BMMs grown on 1, 10, or 20kPa gels prior to incubation with fl-oxLDL, uptake of fl-oxLDL was lowest in BMMs grown on 1kPa gels and two-fold higher in BMMs grown on 10kPa and 20kPa gels (Fig 2A). Uptake of fl-acLDL in BMMs grown on 1, 10, or 20kPa gels was also lowest on 1kPa gels; uptake increased in BMMs on 20kPa gels by approximately 17% (Fig 2B). Thus, uptake of both fl-oxLDL and fl-acLDL was significantly increased in BMMs grown on 10kPa versus 1kPa gels, with no further increase on 20kPa gels. We conclude modified LDL uptake is dependent on growth surface stiffness, and is greater at linear stiffnesses that approximate atherosclerotic artery (10-20kPa) compared to that of normal blood vessel (1-2kPa) [3].

### Linear stiffness regulates expression of LOX-1, CD36, and SRb1

Receptor-mediated endocytosis is the dominant mechanism for the uptake of oxLDL and acLDL; thus, we next asked whether linear stiffness of the growth surface regulated expression of key lipoprotein uptake receptors: CD36, SRA (bind oxLDL and acLDL); SRb1 (binds oxLDL, acLDL, and high density lipoprotein); LOX-1 (binds oxLDL), and LDL receptor (LDL-R; binds native LDL) [10, 15, 39].

**Fig 3:**
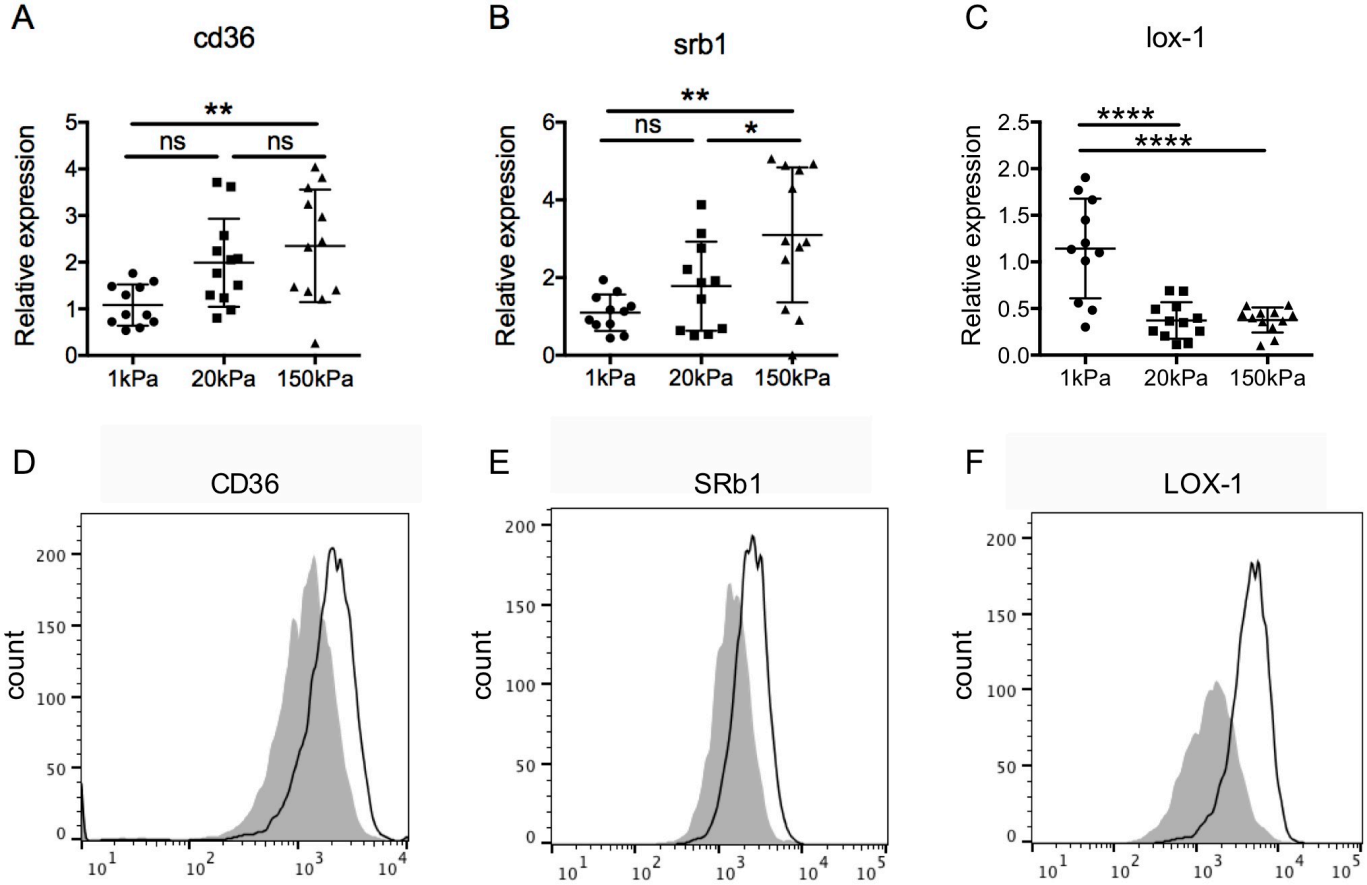
Growth surface stiffness regulates macrophage expression of CD36, SRb1, and LOX1. Primary bone marrow-derived macrophages (BMMs) were grown on fibronectin-coated polyacrylamide gels of the indicated stiffness (kPa; kilopascals) for 24 hours. A-C) Quantitative real-time PCR was used to measure mRNA expression of A) cd36, B) scavenger receptor b1 (srb1), and C) lectin-type oxidized LDL receptor-1 (lox-1). Expression was normalized to 18S mRNA. Graphs are a compilation from 3 independent experiments. Mean +/- standard deviation is shown, and data were analyzed by one-way ANOVA with Tukey’s multiple comparisons test. * p < 0.05, ** p < 0.01. D-F) BMMs were removed from the gels and analyzed by flow cytometry for surface expression of D) CD36, E) SRb1, and F) LOX-1. Data are representative histograms from two independent experiments. Gray shaded = BMMs on 1kPa; black open line = BMMs on 10kPa.

BMMs on 150kPa PA gels expressed higher levels of CD36 and SRb1 mRNA than BMMs on 1kPa PA gels (Fig 3A and 3B). While there was a trend for intermediate levels of expression at 20kPa, the difference was only significant for SRb1 (Fig 3B). Unexpectedly, mRNA expression of LOX-1 was approximately two-fold lower in macrophages adapted to PA gels of 20kPa or 150kPa stiffness compared to those on 1kPa PA gels (Fig 3C). No differences in the mRNA expression of LDL-R or SRA were detected (S4A and S4B Fig). There were also no differences in mRNA expression of the cholesterol efflux transporters ATP-binding cassette transporter (ABC)A1 or ABCG1 (S4C and S4D Fig). We conclude that surface linear stiffness regulates mRNA expression of the CD36, SRB1, and LOX-1 scavenger receptors that recognize modified LDLs.

Since uptake of oxLDL differed significantly between BMMs on 1kPa and 10kPa gels, we used flow cytometry to measure surface expression of CD36, SRb1, and LOX-1 on BMMs equilibrated to these two surfaces. Similar to the mRNA expression data, surface expression of CD36 and SRb1 was increased in BMMs on higher stiffness 10kPa gels compared to BMMs on 1kPa gels (Fig 3D and 3E). Interestingly, despite lower mRNA expression on higher stiffness (20kPa and 150kPa) gels, surface expression of LOX-1 was increased in BMMs equilibrated to 10kPa gels (Fig 3F) compared to 1kPa gels. We conclude that increased surface stiffness (10kPa vs 1kPa) signals macrophages to upregulate surface expression of the lipid uptake receptors CD36, SRb1, and LOX-1, which correlates with increased uptake of oxLDL and acLDL.

### Macrophage uptake of oxLDL requires functional FAK

**Fig 4:**
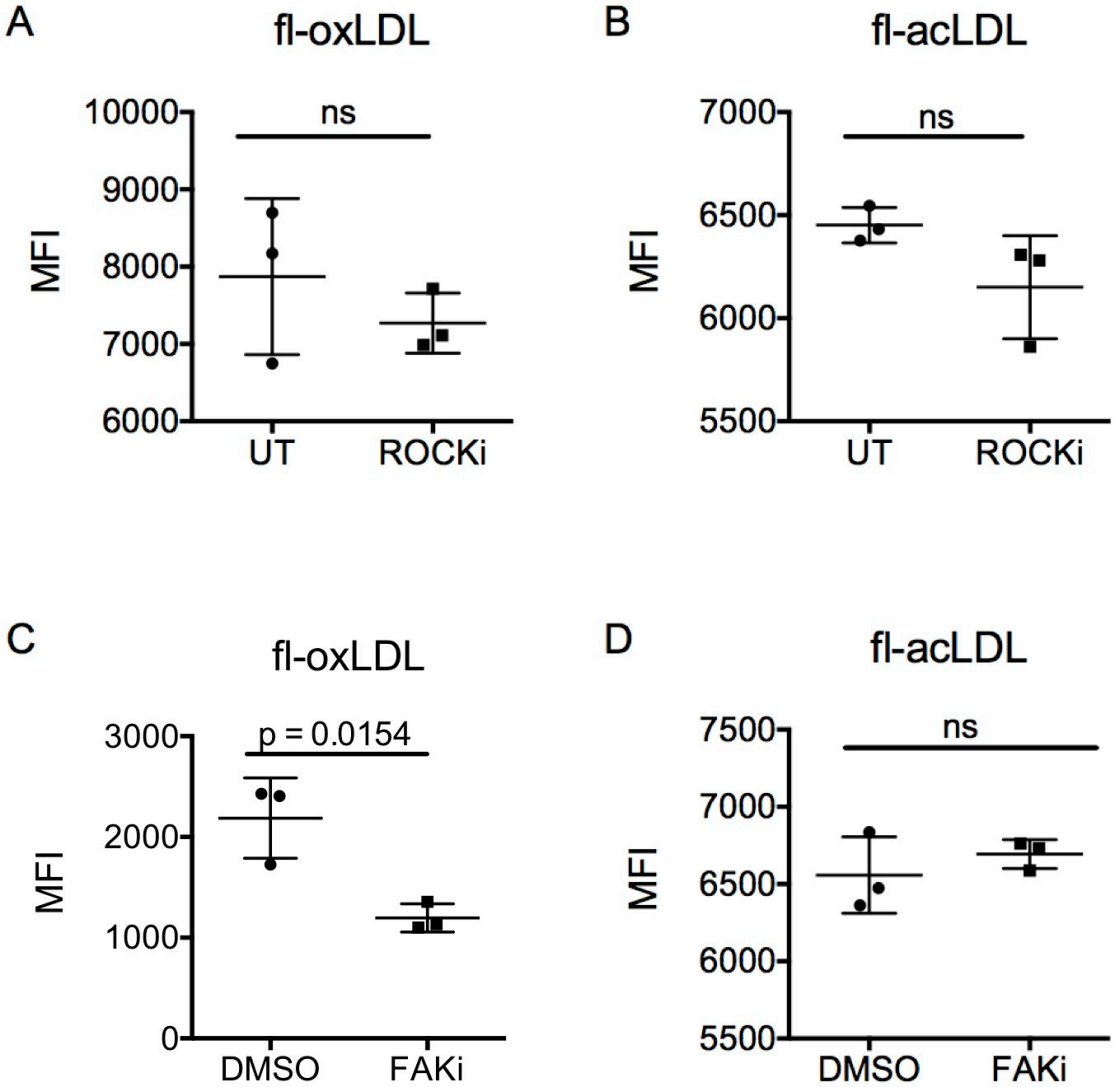
Uptake of oxLDL and acLDL are independent of ROCK1/2; maximal uptake of oxLDL requires FAK. Primary bone marrow-derived macrophages (BMMs) were grown on fibronectin-coated glass for 18 hours. A) BMMs were treated with the rho-associated coiled coil kinase (ROCK)1/2 inhibitor, Y-27632 (10μM, ROCKi), or vehicle control for 1 hour, followed by a 4-hour incubation with fluorescently labeled oxidized LDL (fl-oxLDL; 2μg/ml). BMMs were removed and fluorescence was measured by flow cytometry. B) As in (A), except with fluorescently labeled acetylated LDL (fl-acLDL; 2μg/ml). C) As in (A), except BMMs were pretreated with the focal adhesion kinase (FAK) inhibitor, PF-573228 (10μM, FAKi), or vehicle control and fl-oxLDL. D) As in (C), except with fl-acLDL. Each point is the median fluorescent intensity (10,000 events) from one biologic replicate. Mean +/- standard deviation is shown, and results were analyzed by the unpaired t-test. Data are representative of a minimum of three independent experiments.

We recently showed that TLR4-induced TNFα secretion is higher in macrophages equilibrated to 1kPa gels compared to stiff gels, and that mechanotransduction via ROCK1/2 attenuates TLR signaling and cytokine release [26]. Here, we asked whether uptake of either fl-oxLDL or fl-acLDL was also dependent on signaling through ROCK1/2. BMMs were plated on fibronectin-coated glass coverslips, an extremely high stiffness where mechanotransduction signaling would be expected to be highest. At this extreme stiffness, we found that pharmacologic inhibition of ROCK1/2 for had no effect on the uptake of either fl-oxLDL or fl-acLDL in BMMs (Fig 4A and 4B). When mechanotransduction signaling was more broadly inhibited with an inhibitor of FAK, a major signaling hub upstream of ROCK1/2, uptake of fl-oxLDL was decreased (Fig 4C). In contrast, inhibition of FAK had no effect on the uptake of fl-acLDL (Fig 4D). Thus, we conclude that FAK, but not ROCK, regulates oxLDL uptake, and that neither kinase is important for acLDL uptake.

### Inhibition of TRPV4 enhances macrophage uptake of acLDL

**Fig 5:**
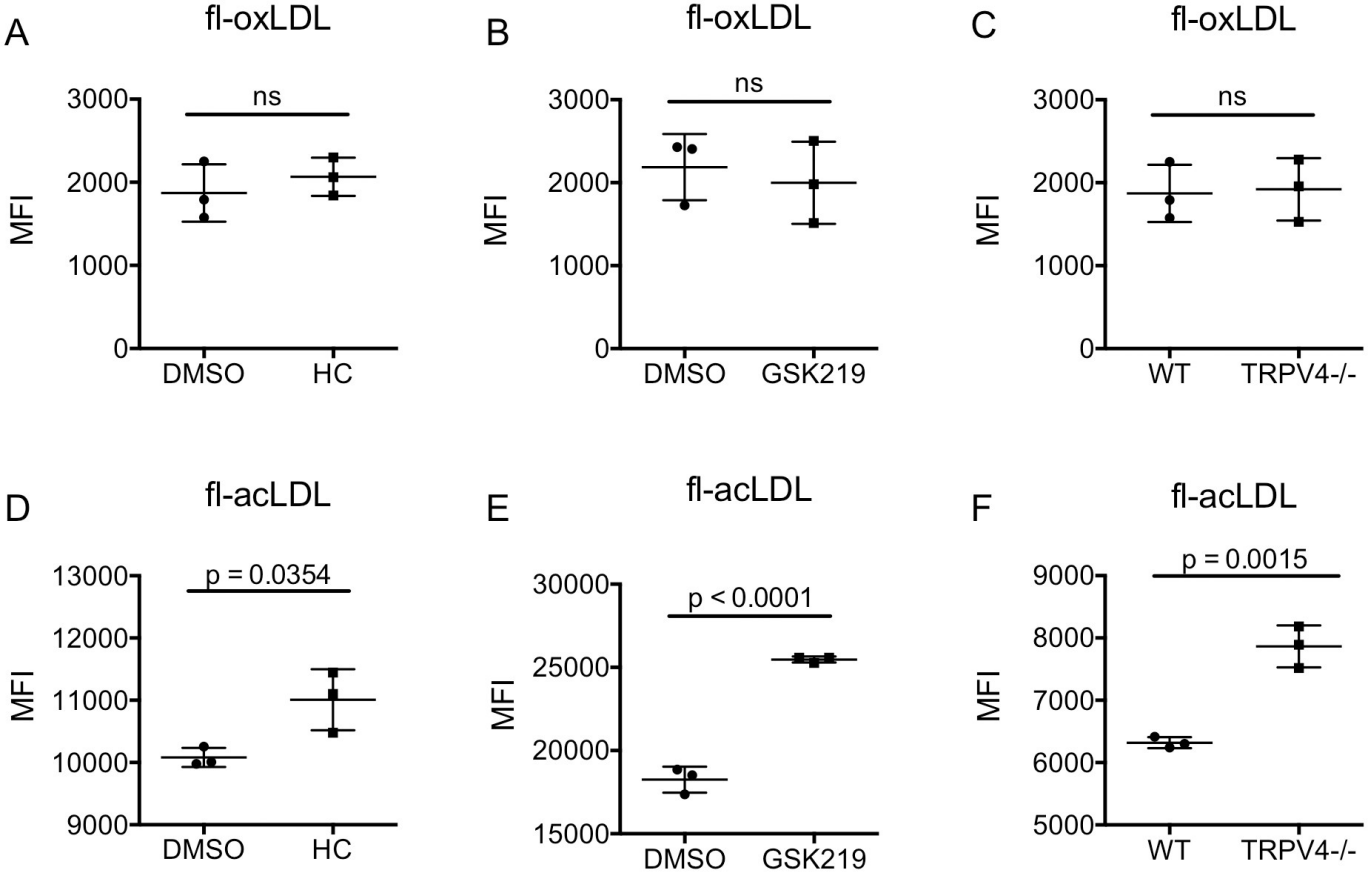
Decreased TRPV4 activity enhances the uptake of acLDL, but not oxLDL. Primary bone marrow-derived macrophages (BMMs) were grown on fibronectin-coated glass for 18 hours. A) BMMs were treated with the transient receptor potential vanilloid (TRPV4) inhibitors: HC-067047 (7.5μM, HC) or vehicle control (DMSO) for 1 hour, followed by a 4-hour incubation with fluorescently labeled oxidized LDL (fl-oxLDL; 2μg/ml). BMMs were removed and fluorescence was measured by flow cytometry. B) As in (A) except with GSK2193874 (5μM, GSK219). C) BMMs isolated from wild type C57B/6 (WT) or TRPV4-/- mice were incubated with fl-oxLDL (2μg/ml) for 4 hours. Cells were removed and fluorescence was measured by flow cytometry. D-F) As in (A-C, respectively), except with fluorescently labeled acetylated LDL (fl-acLDL; 2μg/ml). Each point is the median fluorescent intensity (10,000 events) from a single biologic replicate. Mean +/- standard deviation is shown, and results were analyzed by the unpaired t-test. Data are representative of a minimum of three independent experiments.

Scheraga et al. showed that the mechanosensitive ion channel, TRPV4, was required for maximal LPS-induced phagocytosis in alveolar and bone marrow derived macrophages [29, 40]. The Rahaman group showed genetic deletion and pharmacologic inhibition of TRPV4 decreased uptake of oxLDL by peritoneal macrophages [41, 42]. Given potential functional differences in peritoneal versus bone marrow-derived macrophages, we next asked whether uptake of oxLDL or acLDL was similarly regulated in bone marrow-derived macrophages. We first measured the uptake of oxLDL and acLDL in the presence of two different inhibitors of TRPV4. HC-067047 is a small molecule inhibitor of TRPV4, with little to no effect on other TRP channels [43]. GSK2193874 is a less selective small molecule inhibitor of TRPV4 that also inhibits TRPV1, TRPA1, TRPC3, TRPC6, and TRPM8 [44, 45]. In contrast to previous studies in peritoneal macrophages, uptake of fl-oxLDL by BMMs was not affected by either TRPV4 inhibitor (Fig 5A and 5B). Unexpectedly, both TRPV4 inhibitors increased fl-acLDL uptake by 10–40% (Fig 5D and 5E). These data support a role for TRPV4 in negatively regulating uptake of acLDL.

To confirm these findings, we measured uptake of fl-oxLDL and fl-acLDL by BMMs derived from WT and TRPV4-/- mice. Similar to acute pharmacologic inhibition of TRPV4, genetic absence of complete TRPV4 had no effect on the uptake of fl-oxLDL in BMMs (Fig 5C). However, uptake of fl-acLDL was increased in TRPV4-/- BMMs compared to WT BMMs (Fig 5F). We conclude that TRPV4 activity limits uptake of acLDL, but not oxLDL, in BMMs.

### TRPV4 mediates lipid uptake independent of stiffness

**Fig 6:**
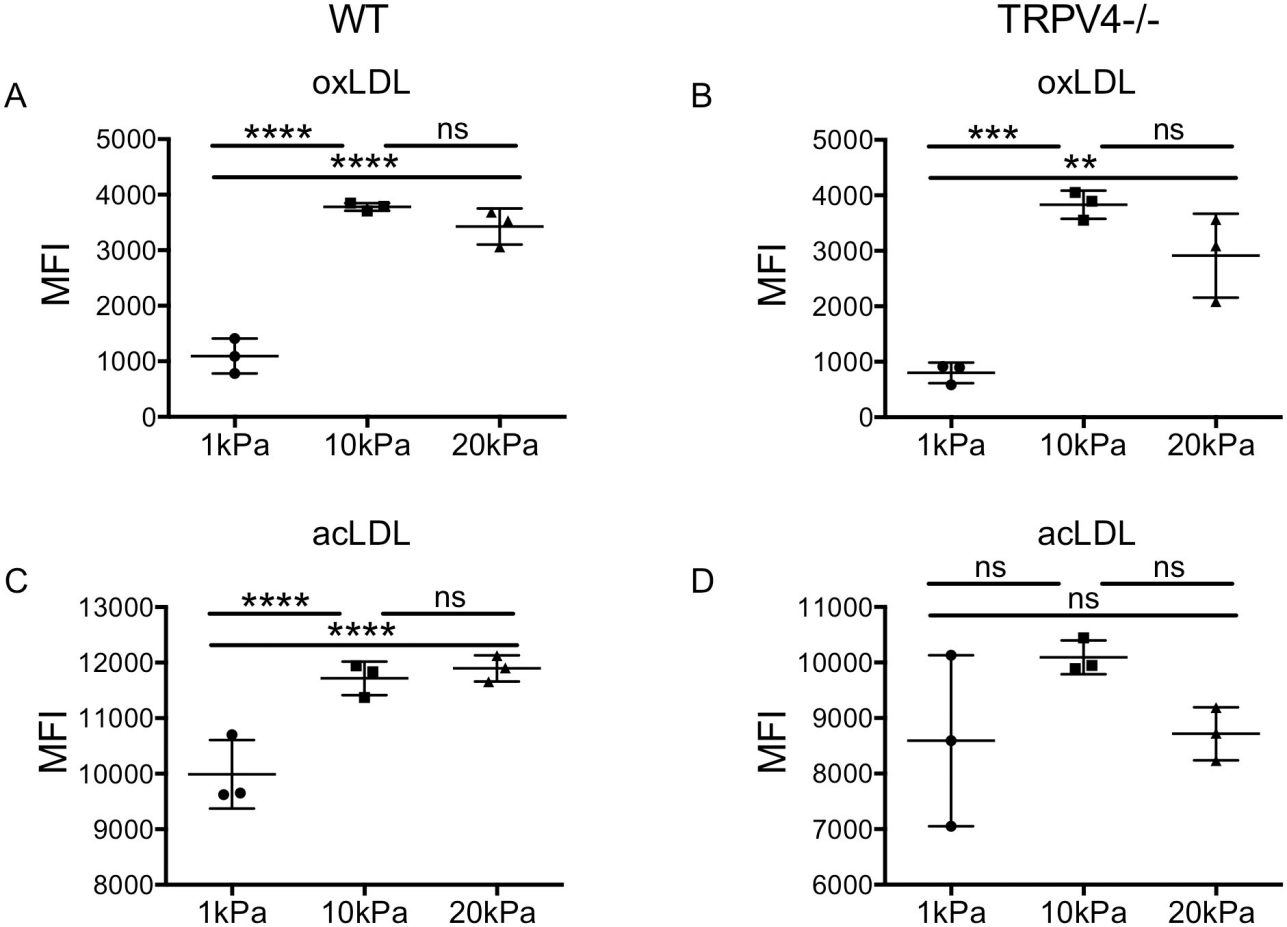
TRPV4 regulates acLDL uptake independently of linear stiffness. A) Primary bone marrow-derived macrophages (BMMs) from wild type C57B/6 mice (WT) were grown on fibronectin-coated 1, 10, and 20kilopascal (kPa) polyacrylamide gels 24 hours prior to incubation with fluorescently labeled oxidized LDL (fl-oxLDL; 2μg/ml) for 4 hours. BMMs were removed and fluorescence was analyzed by flow cytometry. B) As in (A), except with BMMs from TRPV4-/- mice. C) As in (A), except with fluorescently labeled acetylated LDL (fl-acLDL; 2μg/ml). D) As in (C), except with fl-acLDL (2μg/ml). Each point is the median fluorescent intensity (10,000 events) from a one biologic replicate. Mean +/- standard deviation is shown, and results were analyzed by one-way ANOVA with Tukey’s multiple comparisons test. ** p < 0.01, *** p < 0.001, **** p < 0.0001. Graphs are representative of a minimum of two independent experiments.

TRPV4 activity is regulated, in part, by mechanical stimuli, although the precise mechanisms governing this effect are not clear [29, 40]. We reasoned that if mechanoregulation of acLDL uptake occurred via altered TRPV4 activity, the stiffness-dependent differences in macrophage uptake of fl-acLDL, but not fl-oxLDL, would be abrogated in TRPV4-/- BMMs. As predicted, absence of full TRPV4 did not alter the stiffness-dependent increase in uptake of fl-oxLDL (Fig 6A and 6B), supporting the acute inhibition studies and our conclusion that TRPV4 does not regulate oxLDL uptake. In contrast, the stiffness-dependent differences in the uptake of fl-acLDL were abrogated in TRPV4-/- BMMs compared to WT BMMs (Fig 6C and 6D). We conclude that signaling through TRPV4, likely along with other as-yet unidentified mechanotransducers, regulates the uptake of acLDL.

### Basal ROS production and response to oxLDL are linear stiffness-dependent

Reactive oxygen species (ROS) are highly reactive oxygen-containing molecules such as superoxide, hydrogen peroxide, hydroxyl radical, and peroxynitrite [46]. ROS produced by macrophages in the arterial intima induce tissue damage and contribute to the development of atherosclerosis by promoting inflammation and smooth muscle cell proliferation [47–49]. Because oxidative stress has been associated with increased arterial stiffness [50], we next asked whether surface stiffness regulates basal ROS production in BMMs.

**Fig 7:**
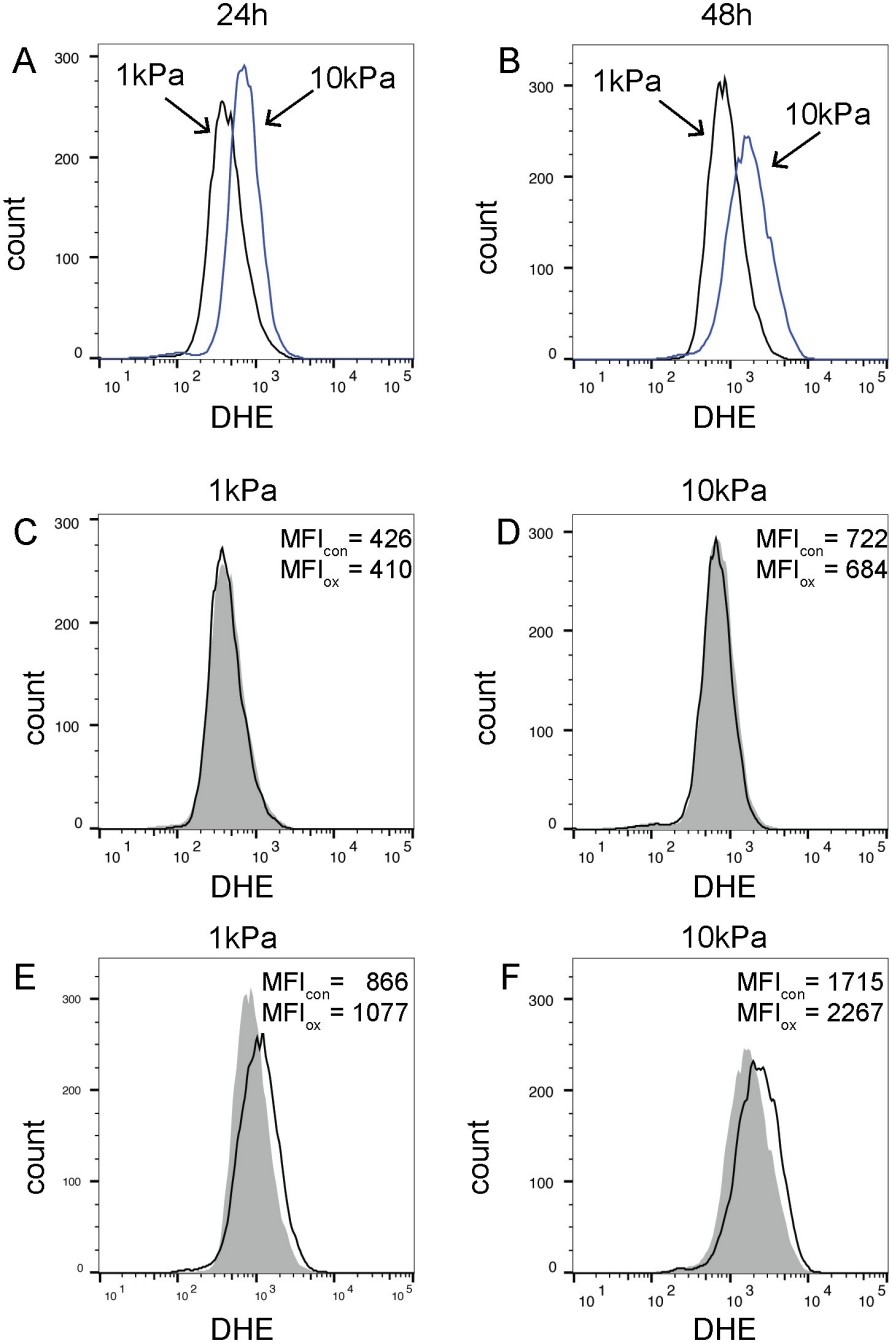
BMM production of reactive oxygen species is increased on stiff versus soft polyacrylamide gels. A, B) Primary bone marrow-derived macrophages (BMMs) were grown on fibronectin-coated 1 or 10kilopascal (kPa) polyacrylamide gels for 24 hours (A) or 48 hours (B), at which time media was replaced with Hank’s buffered saline containing dihydroethidium (DHE, 10μM) for 45 minutes. Cells were washed and removed for analysis by flow cytometry. Black line = BMMs on 1kPa. Blue line = BMMs on 10kPa. C, D) As in (A), except that at 22 hours, media was replaced with new media +/- oxidized LDL (oxLDL; 25μg/ml) for 2 hours prior to incubation with DHE. E, F) As in (B), except that at 24 hours, media was replaced with new media +/- oxLDL (25μg/ml) for 24 hours prior to incubation with DHE. C-F) BMMs not treated with oxLDL (control) are depicted by the gray shaded plot and MFI_con_ (median fluorescent intensity). BMMs treated with oxLDL are depicted by the open black line and MFI_ox_, Data are representative of a minimum of two independent experiments.

We found that basal ROS levels were significantly higher in unstimulated BMMs equilibrated for 24 hours on 10kPa PA gels compared to 1kPa PA gels (Fig 7A). After 48 hours, ROS production was increased by BMMs on both stiffnesses, but the increased production on 10kPa was more pronounced (Fig 7B). Equilibration of BMMs on fibronectin-coated glass coverslips resulted in an even higher basal ROS production (S5 Fig); however, this stiffness is well beyond the normal physiological stiffness of tissues [51, 52].

Because oxLDL is known to induce of ROS production by multiple cell types, including macrophages [53–56], we next asked whether oxLDL-induced ROS production would be increased in BMMs on 10kPa compared to 1kPa PA gels. oxLDL did not induce an increase in ROS at 2 hours (Fig 7C and 7D) in BMMs on either stiffness. In contrast, after 24 hours, ROS production in oxLDL-treated BMMs on both stiffnesses was increased compared to baseline and was higher in BMMs on 10kPa than on 1kPa (Fig 7E and 7F). We conclude that increased stiffness enhances basal and oxLDL-induced production of ROS by macrophages.

## Discussion

In this study, we provide evidence that growth surface stiffness regulates macrophage uptake of both oxLDL and acLDL, two lipoproteins critical to the pathogenesis of atherosclerosis. Stiffness-dependent increases in lipid uptake correlated with increased surface expression of the scavenger receptors CD36, SRb1, and LOX-1. Pharmacologic inhibition of a major mechanosignaling hub, FAK, decreased uptake of oxLDL, but did not affect uptake of acLDL. Instead, we found that uptake of acLDL is negatively regulated by the mechanosensitive ion channel, TRPV4. Additionally, we show that ROS generation in both untreated and oxLDL-treated macrophages was greater on higher stiffness surfaces.

Previous studies in LPS-primed macrophages reported enhanced phagocytosis in macrophages equilibrated to stiff versus soft growth surfaces [28, 29]. Here, we found that in unprimed macrophages, stiffness did not alter the degree of phagocytosis of silica beads or multiple bioparticles. Since activation of macrophages by LPS and other ligands regulates phagocytosis [57–59], and since TLR signaling and cytokine release in response to LPS is regulated by mechanical cues [26, 28, 60, 61], we speculate that the stiffness-dependent differences in phagocytosis observed in LPS-primed macrophage may, in fact, reflect mechanoregulation of TLR signaling with downstream effects on phagocytosis.

Although phagocytosis in unprimed macrophages did not depend on stiffness cues, uptake of oxLDL and acLDL, and the production of ROS were all increased when unprimed macrophages were equilibrated to stiff versus soft surfaces. These findings complement recent microscopy studies that demonstrated increased internalization of oxLDL by murine peritoneal macrophages grown on 8kPa hydrogels compared to 0.5kPa hydrogels [41, 42]. If uptake of lipoproteins is similarly enhanced by stiffness in vivo, an important implication of these findings is that early changes in vessel wall stiffness that occur prior to overt atherosclerosis may signal macrophages to take up more oxLDL and acLDL, thus promoting foam cell formation and driving the progression of disease [2]. Foam cell formation promotes inflammation which, in turn, drives fibrotic changes and the subsequent increased vessel wall [15, 62]. Thus, macrophage mechanotransduction signaling may be important to the feed-forward phenomenon that drives the progression of atherosclerosis, raising the possibility of a potential novel target for intervention.

Increased production of ROS also likely contributes to the feed-forward progression of atherosclerosis. Oxidative stress and ROS are thought to promote oxidation of oxLDL, which in turn, stimulates generation of more ROS [63, 64]. Here, we show that both basal and oxLDL-induced ROS production were increased in macrophages grown on stiff surfaces. Moreover, it appears that the stiffness of the underlying substrate had greater impact on ROS production than did the presence of oxLDL. Oxidative stress has previously been associated with increased arterial stiffness [50], and our findings add to the growing body of evidence implicating mechanical signaling in the regulation of ROS production. Analogous studies have demonstrated that cell stretching enhances ROS production in endothelial cells, cardiomyocytes, and vascular smooth muscle cells [65–67]. Shear stress and altered flow dynamics also stimulate ROS production by endothelial cells [68, 69]. For example, porcine aortic endothelial cells activated by phorbol 12-myristate 13-acetate (PMA) generated more ROS on stiff versus soft polyacrylamide gels [70]. These data raise the idea that increased arterial wall stiffness that precedes the development of the plaque alters mechanotransduction pathways in macrophages and other cells to favor the production of pro-inflammatory ROS that contribute to the oxidation of intimal LDL, thus propelling the progression of atherosclerosis [63, 64].

Although the mechanoregulatory mechanisms underlying these observations are undoubtedly complex, we have begun to tease out several key players. We observed stiffness-dependent increases in mRNA and surface expression of two lipid uptake receptors: CD36 and SRb1. CD36 is a classic scavenger receptor, with affinity for both acLDL and oxLDL [9]. SRb1 has sequence homology with CD36; however its function in lipid handling is more complex since it mediates the uptake of both acLDL and oxLDL, as well as the efflux of cholesterol to high density lipoprotein (HDL) for subsequent transport to the liver [71]. The precise mechanisms mediating the stiffness-dependent transcriptional upregulation of CD36 and SRb1 remain to be determined; candidate mechanosensitive transcriptional regulators include Yes-associated protein/transcriptional coactivator with PDZ-binding motif (YAP/TAZ), myocardin-related transcription factor–A, nuclear factor κB, and members of the Kruppel-like factor family, (KLF2 and KLF4) [72–75].

Mechanoregulation of the surface expression of the oxLDL-specific uptake receptor, Lox-1, appears to be more complex than that of CD36 and Srb1. Surface expression of LOX-1 protein was higher in macrophages on stiff gels, yet LOX-1 mRNA expression was lower. These findings strongly suggest that mechanotransduction signaling pathways regulate Lox-1 expression at multiple points, consistent with other studies [75–77].

Under conditions of extreme mechanical stiffness (e.g. glass; ~2-4GPa), we found that the classic mechanotransduction mediator, FAK, is required for uptake of oxLDL, but not of acLDL. The downstream rho/ROCK pathway does not appear to be involved in the uptake of either oxLDL or acLDL. Further investigation is needed to determine precisely which of multiple FAK downstream signaling cascades regulates the uptake of oxLDL. A prime candidate is Rac1, which is activated by stiffness-dependent FAK-p130CAS signaling to promote entry into the cell cycle [78]. In addition to its role in cellular proliferation, Rac1 is involved in the uptake of particles colocalizing with F-actin in the membrane ruffles of cholesterol-loaded macrophages [79]. Recent studies have reported decreased uptake of oxLDL in Rac1-/- BMMs, although the mechanism underlying this observation was not determined [80, 81]. One group reported increased CD36 and decreased COX2 and ABCG1 in Rac-/- compared to WT BMMs, and hypothesized that Rac1 may be important in CD36 localization [80]. A direct link connecting substrate stiffness, Rac1 signaling, and lipid uptake has yet to be established. Another candidate pathway downstream of FAK is the cdc42-mediated pathway, which is activated in macrophages on stiff surfaces, and involved in both phagocytosis and Akt3-regulated LDL pinocytosis [28, 59, 82].

We show here for the first time that TRPV4 negatively regulates uptake of acLDL in macrophages equilibrated to glass. TRPV4 is a calcium channel and plays a protective role during atherosclerosis by regulating monocyte recruitment to the forming plaque. Mice treated with a TRPV4 agonist had lower atherosclerotic plaque formation on a high fat diet compared to control; however effects of TRPV4 on macrophage uptake of LDL were not assessed [83]. Our data now show that uptake of both acLDL and oxLDL were augmented when TRPV4 was inhibited. We speculate that signaling events, that may be in part dependent on calcium, are responsible for negatively regulating LDL uptake, and that treatment with TRPV4 inhibitor reduces those negative signals thereby enhancing LDL uptake. Of note, the stiffness-dependent differences in acLDL uptake were blunted in TRPV4-/- BMMs, implicating TRPV4 in the mechanoregulation of acLDL uptake. Since acLDL stimulates cytokine expression and release by macrophages [84–86], limiting the uptake of acLDL via TRPV4 signaling has the potential to reduce inflammation in atherosclerotic lesions.

In contrast, we show that neither acute pharmacologic inhibition nor genetic deletion of TRPV4 had an effect on the uptake of oxLDL in WT BMMs. These findings differ from the Rahaman group, who found that TRPV4 that was critical for the uptake of oxLDL by peritoneal macrophages [41, 42]. Our data indicate that TRPV4 differentially regulates the uptake of acLDL and oxLDL, and in light of findings by the Rahaman group, we speculate that the role of TRPV4 in regulating lipoprotein uptake may be context-dependent. For example, our studies were conducted in primary murine BMMs, whereas the Rahaman group used murine peritoneal macrophages and RAW264.7 cells. In addition to potential strain differences (primary C57B/6 cells versus BALB/c RAW264.7 cells), phenotypic differences and response to oxLDL have been documented in BMM and peritoneal macrophages derived from the same mouse strain (ApoE-/-), and there are differences in the functional activity peritoneal and bone marrow-derived macrophages [87, 88]. Another methodological difference, is that our studies were performed using polyacrylamide gels coated with fibronectin, an ECM protein that is enriched in atherogenic lesions, whereas the previous studies used collagen-coated gels [37, 38, 42]. Different ECM proteins engage different integrin receptor subsets, leading to differential activation of downstream signaling pathways [89–98]. Differences in methodology and the degree of oxidation of LDL particles (e.g. minimally, moderately, or extensively) could also contribute to differences between the two studies [54, 64, 99–103]. Given the well-documented role for TRPV4 in calcium flux, and the important role that calcium flux plays in numerous functions including phagocytosis and pinocytosis [40, 104], it is possible that TRPV4 deficiency alters lipid uptake via altered calcium flux; however, this remains to be definitively demonstrated and may involve an as-yet unrecognized alternative mechanism. Overall, TRPV4 likely plays a complex and multifaceted role in atherosclerosis development and agonism or antagonism with pharmaceutical intervention requires additional study before translation for human therapy.

Atherosclerosis is a complex chronic disease with lesions that are not readily sampled in human patients. Studies in large animal models (e.g. pig and nonhuman primates) are costly and time-consuming, thus most advances have relied on murine models, which require genetic modifications to induce atherosclerotic lesions (e.g. ApoE-/-, ldlr-/-) [105]. The studies described herein build on previous murine studies modeling human atherosclerosis. Potential ways to study these processes in humans include the cell line THP1 derived from monocytic leukemia and primary human monocyte-derived macrophages. Both THP1 cells and human monocytes are non-adherent. THP1 cells require PMA stimulation to induce the adherent macrophage phenotype, and human monocytes much be differentiated for seven days in GM-CSF, in order to study the effects of extracellular stiffness in our model [106]. PMA treatment would make interpretation of these studies challenging since it changes in expression of CD36, a receptor we showed in this paper is also regulated by surface stiffness [107]. Furthermore, compared to primary monocytes, THP1 cells are deficient in CD14, and are thus hyporesponsive to LPS and may result in decreased uptake of minimally oxidized LDL [101, 108, 109]. Thus, additional models or ex vivo studies will be required to define more detailed mechanisms.

In conclusion, our study provides in vitro data linking increased linear stiffness with two key functions critical to the pathogenesis of atherosclerosis: macrophage accumulation of modified LDLs and ROS generation. Well-controlled in vivo studies that completely isolate the effects of mechanics from the biochemical milieu are not yet possible; thus in vitro studies such those described herein are critical to understanding the role of physical cues in the regulation of macrophage function. Unraveling the role of mechanotransduction in lipid handling could have ramifications in our understanding, management, and prevention of atherosclerosis.

## Supporting information

S1 Fig Phagocytosis after five minutes in unprimed macrophages is independent of linear stiffness. Primary bone marrow-derived macrophages BMMs were grown on fibronectin-coated 1, 20, 150 kilopascal (kPa) polyacrylamide gels or fibronectin-coated glass for 24h. BMMs were incubated with fluorescently-labeled (A-B) silica beads, (C-D) *E*.*coli*, (E-F) *S*.*aureus*, or (G-H) Zymosan A for 5min, fixed in 3% paraformaldehyde, stained with phalloidin (F-actin), and imaged by epifluorescence microscopy. (A, C, E, G) show the percentage of BMMs on each of the substrates that phagocytosed at least one particle. Mean +/- SD from three independent experiments is shown. Data were analyzed by one-way ANOVA with Tukey’s multiple comparisons test; no significant differences were reported. (B, D, F, H) The number of internalized particles per cell from a minimum of 50 BMMs per condition in three independent experiments were quantified. Results were analyzed by the Kruskal-Wallis test.
(TIF)

S2 Fig Representative epifluorescent images used to quantify phagocytosis in Fig 1 and S1 Fig. Primary bone marrow-derived macrophages BMMs were grown on fibronectin-coated 1, 20, 150 kilopascal (kPa) polyacrylamide gels or fibronectin-coated glass for 24h. BMMs were incubated with fluorescently-labeled (A-D) silica beads, (E-H) *E*.*coli*, (I-L) *S*.*aureus*, or (M-P) Zymosan A for 30min, fixed in 3% paraformaldehyde, stained with phalloidin (F-actin), and imaged with an Axio Imager M1 microscope. Original objective magnification 63x, scale bar = 10μm.
(TIFF)

S3 Fig Uptake of oxLDL and acLDL is time- and dose- dependent. Primary bone marrow-derived macrophages (BMMs) were grown on fibronectin-coated glass for 24 hours, treated as indicated, and then removed into a single cell suspension for analysis by flow cytometry. Treatments were as follows: A) fluorescently labeled oxidized LDL (fl-oxLDL) at final concentrations of 0.5, 1, 2, 4, 8 μg/ml) for 4 hours; B) fluorescently labeled acetylated LDL (fl-acLDL) at final concentration of 0.5, 1, 2, 6, 10 μg/ml) for 4 hours. C) fl-oxLDL (2μg/ml) for 0.5, 1, 2, 4, 6, 24 hours; or D) fl-acLDL (2μg/ml) for 0.5, 1, 2, 4, 24 hours. Darker shades of red correspond to higher concentrations of fl-oxLDL (A) or fl-acLDL (B). Darker shades of blue correspond to longer incubation times with fl-oxLDL (C) or fl-acLDL (D). Data are representative of a minimum of two independent experiments.
(TIF)

S4 Fig Linear stiffness does not regulate mRNA expression of sra, ldl-r, abca1, or abcg1. Primary bone marrow-derived macrophages (BMMs) were grown on fibronectin-coated 1, 20, 150 kilopascal (kPa) polyacrylamide gels for 24 hours. Relative gene expression was quantified by quantitative PCR using the ΔΔCt method and 18S for normalization. A) scavenger receptor A (sra); B) LDL receptor (ldl-r); C) ATP binding cassette transporter A1 (abca1); D) abcg1. Results were analyzed by one-way ANOVA with Tukey’s multiple comparisons test.
(TIF)

S5 Fig Basal production of reactive oxygen species is dependent on linear stiffness. Primary bone marrow-derived macrophages (BMMs) were grown on fibronectin-coated 1 or 10 kilopascal (kPa) polyacrylamide gels or glass for 24 hours. Media was replaced with Hank’s buffered saline (UT, grey-filled) or dihydroethidium (DHE, 10μM) in Hank’s buffered saline for 45 minutes. Cells were washed and removed for analysis by flow cytometry. B) As in (A), except that BMMs were grown on each surface for 48 hours prior to treatment with and without DHE. Gray shaded = BMMs without DHE (UT); Open black line = BMMs on 1kPa incubated with DHE. Open blue line = BMMs on 10kPa incubated with DHE. Open red line = BMMs on glass incubated with DHE. Data are representative of a minimum of two independent experiments.
(TIF)

## References

[1] Gisterå A, Hansson GK. The immunology of atherosclerosis. Nat Rev Nephrol. 2017;13: 368–380. doi: 10.1038/nrneph.2017.51 28392564

[2] Gotschy A, Bauer E, Schrodt C, Lykowsky G, Ye Y-X, Rommel E, et al. Local arterial stiffening assessed by MRI precedes atherosclerotic plaque formation. Circ Cardiovasc Imaging. 2013;6: 916–23. doi: 10.1161/CIRCIMAGING.113.000611 24100044

[3] Tracqui P, Broisat A, Toczek J, Mesnier N, Ohayon J, Riou L. Mapping elasticity moduli of atherosclerotic plaque in situ via atomic force microscopy. J Struct Biol. 2011;174: 115–23. doi: 10.1016/j.jsb.2011.01.010 21296163

[4] Palombo C, Kozakova M. Arterial stiffness, atherosclerosis and cardiovascular risk: Pathophysiologic mechanisms and emerging clinical indications. Vascul Pharmacol. 2016;77: 1–7. doi: 10.1016/j.vph.2015.11.083 26643779

[5] Jovinge S, Ares MP, Kallin B, Nilsson J. Human monocytes/macrophages release TNF-alpha in response to Ox-LDL. Arterioscler Thromb Vasc Biol. 1996;16: 1573–9. doi: 10.1161/01.atv.16.12.1573 8977464

[6] Brown MS, Goldstein JL. Receptor-mediated endocytosis: insights from the lipoprotein receptor system. Proc Natl Acad Sci U S A. 1979;76: 3330–7. doi: 10.1073/pnas.76.7.3330 226968 PMC383819

[7] Moore KJ, Sheedy FJ, Fisher EA. Macrophages in atherosclerosis: a dynamic balance. Nat Rev Immunol. 2013;13: 709–21. doi: 10.1038/nri3520 23995626 PMC4357520

[8] McLaren JE, Michael DR, Ashlin TG, Ramji DP. Cytokines, macrophage lipid metabolism and foam cells: implications for cardiovascular disease therapy. Prog Lipid Res. 2011;50: 331–47. doi: 10.1016/j.plipres.2011.04.002 21601592

[9] Febbraio M, Silverstein RL. CD36: Implications in cardiovascular disease. Int J Biochem Cell Biol. 2007;39: 2012–2030. doi: 10.1016/j.biocel.2007.03.012 17466567 PMC2034445

[10] Moriwaki H, Kume N, Sawamura T, Aoyama T, Hoshikawa H, Ochi H, et al. Ligand specificity of LOX-1, a novel endothelial receptor for oxidized low density lipoprotein. Arterioscler Thromb Vasc Biol. 1998;18: 1541–7. doi: 10.1161/01.atv.18.10.1541 9763524

[11] Shen W-J, Azhar S, Kraemer FB. SR-B1: A Unique Multifunctional Receptor for Cholesterol Influx and Efflux. Annu Rev Physiol. 2018;80: 95–116. doi: 10.1146/annurev-physiol-021317-121550 29125794 PMC6376870

[12] Sun B, Boyanovsky BB, Connelly MA, Shridas P, van der Westhuyzen DR, Webb NR. Distinct mechanisms for OxLDL uptake and cellular trafficking by class B scavenger receptors CD36 and SR-BI. J Lipid Res. 2007;48: 2560–2570. doi: 10.1194/jlr.M700163-JLR200 17876058

[13] Acton SL, Scherer PE, Lodish HF, Krieger M. Expression cloning of SR-BI, a CD36-related class B scavenger receptor. J Biol Chem. 1994;269: 21003–9. 7520436

[14] Shibata N, Glass CK. Regulation of macrophage function in inflammation and atherosclerosis. J Lipid Res. 2009;50: S277–S281. doi: 10.1194/jlr.R800063-JLR200 18987388 PMC2674700

[15] Moore KJ, Sheedy FJ, Fisher E a. Macrophages in atherosclerosis: a dynamic balance. Nat Rev Immunol. 2013;13: 709–21. doi: 10.1038/nri3520 23995626 PMC4357520

[16] Woollard KJ, Geissmann F. Monocytes in atherosclerosis: subsets and functions. Nat Rev Cardiol. 2010;7: 77–86. doi: 10.1038/nrcardio.2009.228 20065951 PMC2813241

[17] Mantovani A, Garlanda C, Locati M. Macrophage diversity and polarization in atherosclerosis: a question of balance. Arterioscler Thromb Vasc Biol. 2009;29: 1419–23. doi: 10.1161/ATVBAHA.108.180497 19696407

[18] Williams HJ, Fisher EA, Greaves DR. Macrophage Differentiation and Function in Atherosclerosis: Opportunities for Therapeutic Intervention? J Innate Immun. 2012;4: 498–508. doi: 10.1159/000336618 22572544 PMC3598573

[19] Kothapalli D, Liu S-L, Bae YH, Monslow J, Xu T, Hawthorne E a, et al. Cardiovascular protection by ApoE and ApoE-HDL linked to suppression of ECM gene expression and arterial stiffening. Cell Rep. 2012;2: 1259–71. doi: 10.1016/j.celrep.2012.09.018 23103162 PMC3535179

[20] Kaess BM, Rong J, Larson MG, Hamburg NM, Vita JA, Levy D, et al. Aortic Stiffness, Blood Pressure Progression, and Incident Hypertension. JAMA. 2012;308. doi: 10.1001/2012.jama.10503 22948697 PMC3594687

[21] Laurent S, Boutouyrie P, Asmar R, Gautier I, Laloux B, Guize L, et al. Aortic Stiffness Is an Independent Predictor of All-Cause and Cardiovascular Mortality in Hypertensive Patients. Hypertension. 2001;37. doi: 10.1161/01.hyp.37.5.1236 11358934

[22] Weisbrod RM, Shiang T, Al Sayah L, Fry JL, Bajpai S, Reinhart-King CA, et al. Arterial Stiffening Precedes Systolic Hypertension in Diet-Induced ObesityNovelty and Significance. Hypertension. 2013;62. 10.1161/HYPERTENSIONAHA.113.01744 PMC3951434 24060894

[23] Huynh J, Nishimura N, Rana K, Peloquin JM, Califano JP, Montague CR, et al. Age-related intimal stiffening enhances endothelial permeability and leukocyte transmigration. Sci Transl Med. 2011;3: 112ra122. doi: 10.1126/scitranslmed.3002761 22158860 PMC3693751

[24] Tse JR, Engler AJ. Preparation of hydrogel substrates with tunable mechanical properties. Curr Protoc Cell Biol. 2010; 1–16. doi: 10.1002/0471143030.cb2701s47 20521229

[25] Pelham RJ, Wang Y-l. Cell locomotion and focal adhesions are regulated by substrate flexibility. Proc Natl Acad Sci. 1997;94: 13661–13665. doi: 10.1073/pnas.94.25.13661 9391082 PMC28362

[26] Gruber E, Heyward C, Cameron J, Leifer C. Toll-like receptor signaling in macrophages is regulated by extracellular substrate stiffness and Rho-associated coiled-coil kinase (ROCK1/2). Int Immunol. 2018;30: 267–278. doi: 10.1093/intimm/dxy027 29800294 PMC5967458

[27] Julian L, Olson MF. Rho-associated coiled-coil containing kinases (ROCK). Small GTPases. 2014. doi: 10.4161/sgtp.29846 25010901 PMC4114931

[28] Patel NR, Bole M, Chen C, Hardin CC, Kho AT, Mih J, et al. Cell Elasticity Determines Macrophage Function. PLoS One. 2012;7: e41024. doi: 10.1371/journal.pone.0041024 23028423 PMC3445606

[29] Scheraga RG, Abraham S, Niese KA, Southern BD, Grove LM, Hite RD, et al. TRPV4 Mechanosensitive Ion Channel Regulates Lipopolysaccharide-Stimulated Macrophage Phagocytosis. J Immunol. 2016;196: 428–436. doi: 10.4049/jimmunol.1501688 26597012 PMC4684994

[30] Yates RM, Russell DG. Real-Time Spectrofluorometric Assays for the Lumenal Environment of the Maturing Phagosome. In: Deretic V, editor. Autophagosome and Phagosome. Totowa, NJ: Humana Press; 2008. pp. 311–325. 10.1007/978-1-59745-157-4_20 PMC2759531 18425459

[31] Zhang DX, Mendoza SA, Bubolz AH, Mizuno A, Ge Z-D, Li R, et al. Transient receptor potential vanilloid type 4-deficient mice exhibit impaired endothelium-dependent relaxation induced by acetylcholine in vitro and in vivo. Hypertens. 2009;53: 532–8. doi: 10.1161/HYPERTENSIONAHA.108.127100 19188524 PMC2694062

[32] Suzuki M, Mizuno A, Kodaira K, Imai M. Impaired pressure sensation in mice lacking TRPV4. J Biol Chem. 2003;278: 22664–8. doi: 10.1074/jbc.M302561200 12692122

[33] Sones JL, Cha J, Woods AK, Bartos A, Heyward CY, Lob HE, et al. Decidual Cox2 inhibition improves fetal and maternal outcomes in a preeclampsia-like mouse model. JCI Insight. 2016;1: 1–16. doi: 10.1172/jci.insight.75351 27159542 PMC4855694

[34] Xavier AM, Ludwig RG, Nagai MH, de Almeida TJ, Watanabe HM, Hirata MY, et al. CD36 is expressed in a defined subpopulation of neurons in the olfactory epithelium. Sci Rep. 2016;6: 25507. doi: 10.1038/srep25507 27145700 PMC4857125

[35] Crucet M, Wüst SJA, Spielmann P, Lüscher TF, Wenger RH, Matter CM. Hypoxia enhances lipid uptake in macrophages: Role of the scavenger receptors Lox1, SRA, and CD36. Atherosclerosis. 2013;229: 110–117. doi: 10.1016/j.atherosclerosis.2013.04.034 23706521

[36] Schrijvers DM, De Meyer GRY, Herman AG, Martinet W. Phagocytosis in atherosclerosis: Molecular mechanisms and implications for plaque progression and stability. Cardiovasc Res. 2007;73: 470–480. doi: 10.1016/j.cardiores.2006.09.005 17084825

[37] Feaver RE, Gelfand BD, Wang C, Schwartz MA, Blackman BR. Atheroprone hemodynamics regulate fibronectin deposition to create positive feedback that sustains endothelial inflammation. Circ Res. 2010;106: 1703–1711. doi: 10.1161/CIRCRESAHA.109.216283 20378855 PMC2891748

[38] Rohwedder I, Montanez E, Beckmann K, Bengtsson E, Dunér P, Nilsson J, et al. Plasma fibronectin deficiency impedes atherosclerosis progression and fibrous cap formation. EMBO Mol Med. 2012;4: 564–76. doi: 10.1002/emmm.201200237 22514136 PMC3407945

[39] Krieger M. Scavenger receptor class b type I is a multiligand hdl receptor that influences diverse physiologic systems. J Clin Invest. 2001;108: 793–797. doi: 10.1172/JCI14011 11560945 PMC200944

[40] White JPM, Cibelli M, Urban L, Nilius B, McGeown JG, Nagy I. TRPV4: Molecular Conductor of a Diverse Orchestra. Physiol Rev. 2016;96: 911–973. doi: 10.1152/physrev.00016.2015 27252279

[41] Gupta N, Goswami R, Alharbi MO, Biswas D, Rahaman SO. TRPV4 is a regulator in P. gingivalis lipopolysaccharide- induced exacerbation of macrophage foam cell formation. 2019;7. doi: 10.14814/phy2.14069 30980509 PMC6461712

[42] Goswami R, Merth M, Sharma S, Alharbi MO, Aranda-Espinoza H, Zhu X, et al. TRPV4 calcium-permeable channel is a novel regulator of oxidized LDL-induced macrophage foam cell formation. Free Radic Biol Med. 2017;110: 142–150. doi: 10.1016/j.freeradbiomed.2017.06.004 28602913

[43] Everaerts W, Zhen X, Ghosh D, Vriens J, Gevaert T, Gilbert JP. Inhibition of the cation channel TRPV4 improves bladder function in mice and rats with cyclophosphamide-induced cystitis. 2010; 107:19084–9 www.pnas.org/cgi/doi/10.1073/pnas.1005333107 10.1073/pnas.1005333107 PMC2973867 20956320

[44] Cheung M, Bao W, Behm DJ, Brooks CA, Bury MJ, Dowdell SE, et al. Discovery of GSK2193874: An Orally Active, Potent, and Selective Blocker of Transient Receptor Potential Vanilloid 4. 2017; 549–554. doi: 10.1021/acsmedchemlett.7b00094 28523109 PMC5430398

[45] Thorneloe KS, Cheung M, Bao W, Alsaid H, Lenhard S, Jian M-Y, et al. An Orally Active TRPV4 Channel Blocker Prevents and Resolves Pulmonary Edema Induced by Heart Failure. Sci Transl Med. 2012;4: 159ra148–159ra148. doi: 10.1126/scitranslmed.3004276 23136043

[46] Ray PD, Huang B-W, Tsuji Y. Reactive oxygen species (ROS) homeostasis and redox regulation in cellular signaling. Cell Signal. 2012;24: 981–90. doi: 10.1016/j.cellsig.2012.01.008 22286106 PMC3454471

[47] Rao GN, Berk BC. Active oxygen species stimulate vascular smooth muscle cell growth and proto-oncogene expression. Circ Res. 1992;70: 593–9. doi: 10.1161/01.res.70.3.593 1371430

[48] Forrester SJ, Kikuchi DS, Hernandes MS, Xu Q, Griendling KK. Reactive Oxygen Species in Metabolic and Inflammatory Signaling. Circ Res. 2018;122: 877–902. doi: 10.1161/CIRCRESAHA.117.311401 29700084 PMC5926825

[49] Vendrov AE, Hakim ZS, Madamanchi NR, Rojas M, Madamanchi C, Runge MS. Atherosclerosis is attenuated by limiting superoxide generation in both macrophages and vessel wall cells. Arterioscler Thromb Vasc Biol. 2007;27: 2714–2721. doi: 10.1161/ATVBAHA.107.152629 17823367

[50] Patel RS, Al Mheid I, Morris AA, Ahmed Y, Kavtaradze N, Ali S, et al. Oxidative stress is associated with impaired arterial elasticity. Atherosclerosis. 2011;218: 90–95. doi: 10.1016/j.atherosclerosis.2011.04.033 21605864 PMC4059070

[51] Butcher DT, Alliston T, Weaver VM. A tense situation: forcing tumour progression. Nat Rev Cancer. 2009;9: 108–122. doi: 10.1038/nrc2544 19165226 PMC2649117

[52] Inaba S, Fujino S, Morinaga K. Young’s Modulus and Compositional Parameters of Oxide Glasses. J Am Ceram Soc. 2004;82: 3501–3507. doi: 10.1111/j.1151-2916.1999.tb02272.x

[53] Lara-Guzmán OJ, Gil-Izquierdo Á, Medina S, Osorio E, Álvarez-Quintero R, Zuluaga N, et al. Oxidized LDL triggers changes in oxidative stress and inflammatory biomarkers in human macrophages. Redox Biol. 2018;15: 1–11. doi: 10.1016/j.redox.2017.11.017 29195136 PMC5723280

[54] Bae YS, Lee JH, Choi SH, Kim S, Almazan F, Witztum JL, et al. Macrophages generate reactive oxygen species in response to minimally oxidized low-density lipoprotein: Toll-like receptor 4- and spleen tyrosine kinase-dependent activation of NADPH oxidase 2. Circ Res. 2009;104: 210–218. doi: 10.1161/CIRCRESAHA.108.181040 19096031 PMC2720065

[55] Hsieh CC, Yen MH, Yen CH, Lau YT. Oxidized low density lipoprotein induces apoptosis via generation of reactive oxygen species in vascular smooth muscle cells. Cardiovasc Res. 2001;49: 135–45. doi: 10.1016/s0008-6363(00)00218-2 11121805

[56] Rueckschloss U, Galle J, Holtz J, Zerkowski HR, Morawietz H. Induction of NAD(P)H oxidase by oxidized low-density lipoprotein in human endothelial cells: antioxidative potential of hydroxymethylglutaryl coenzyme A reductase inhibitor therapy. Circulation. 2001;104: 1767–72. doi: 10.1161/hc4001.097056 11591612

[57] Doyle SE, O’Connell RM, Miranda GA, Vaidya SA, Chow EK, Liu PT, et al. Toll-like Receptors Induce a Phagocytic Gene Program through p38. J Exp Med. 2004;199: 81–90. doi: 10.1084/jem.20031237 14699082 PMC1887723

[58] Blander JM, Medzhitov R. Regulation of phagosome maturation by signals from toll-like receptors. Science (80-). 2004;304: 1014–1018. doi: 10.1126/science.1096158 15143282

[59] Kong L, Ge BX. MyD88-independent activation of a novel actin-Cdc42/Rac pathway is required for Toll-like receptor-stimulated phagocytosis. Cell Res. 2008;18: 745–755. doi: 10.1038/cr.2008.65 18542102

[60] Previtera ML, Sengupta A. Substrate stiffness regulates proinflammatory mediator production through TLR4 activity in macrophages. PLoS One. 2015;10. doi: 10.1371/journal.pone.0145813 26710072 PMC4692401

[61] Irwin EF, Saha K, Rosenbluth M, Gamble LJ, Castner DG, Healy KE. Modulus-dependent macrophage adhesion and behavior. J Biomater Sci Polym Ed. 2008;19: 1363–1382. doi: 10.1163/156856208786052407 18854128

[62] Thomas AC, Eijgelaar WJ, Daemen MJAP, Newby AC. Foam Cell Formation In Vivo Converts Macrophages to a Pro-Fibrotic Phenotype. PLoS One. 2015;10: e0128163. doi: 10.1371/journal.pone.0128163 26197235 PMC4510387

[63] Hwang J, Ing MH, Salazar A, Lassègue B, Griendling K, Navab M, et al. Pulsatile versus oscillatory shear stress regulates NADPH oxidase subunit expression: implication for native LDL oxidation. Circ Res. 2003;93: 1225–32. doi: 10.1161/01.RES.0000104087.29395.66 14593003 PMC4433384

[64] Levitan I, Volkov S, Subbaiah P V. Oxidized LDL: diversity, patterns of recognition, and pathophysiology. Antioxid Redox Signal. 2010;13: 39–75. doi: 10.1089/ars.2009.2733 19888833 PMC2877120

[65] Aikawa R, Nagai T, Tanaka M, Zou Y, Ishihara T, Takano H, et al. Reactive Oxygen Species in Mechanical Stress-Induced Cardiac Hypertrophy. Biochem Biophys Res Commun. 2001;289: 901–907. doi: 10.1006/bbrc.2001.6068 11735132

[66] Matsushita H, Lee KH, Tsao PS. Cyclic strain induces reactive oxygen species production via an endothelial NAD(P)H oxidase. J Cell Biochem Suppl. 2001;Suppl 36: 99–106. doi: 10.1002/jcb.1094 11455575

[67] Grote K. Mechanical Stretch Enhances mRNA Expression and Proenzyme Release of Matrix Metalloproteinase-2 (MMP-2) via NAD(P)H Oxidase-Derived Reactive Oxygen Species. Circ Res. 2003;92: 80e–86. doi: 10.1161/01.RES.0000077044.60138.7C 12750313

[68] Raaz U, Toh R, Maegdefessel L, Adam M, Nakagami F, Emrich FC, et al. Hemodynamic regulation of reactive oxygen species: implications for vascular diseases. Antioxid Redox Signal. 2014;20: 914–28. doi: 10.1089/ars.2013.5507 23879326 PMC3924901

[69] Hsieh H-J, Liu C-A, Huang B, Tseng AH, Wang DL. Shear-induced endothelial mechanotransduction: the interplay between reactive oxygen species (ROS) and nitric oxide (NO) and the pathophysiological implications. J Biomed Sci. 2014;21: 3. doi: 10.1186/1423-0127-21-3 24410814 PMC3898375

[70] Urbano RL, Swaminathan S, Clyne AM. Stiff Substrates Enhance Endothelial Oxidative Stress in Response to Protein Kinase C Activation. Appl Bionics Biomech. 2019;2019: 1–14. doi: 10.1155/2019/6578492 31110559 PMC6487160

[71] Acton S, Rigotti A, Landschulz KT, Xu S, Hobbs HH, Krieger M. Identification of scavenger receptor SR-BI as a high density lipoprotein receptor. Science. 1996;271: 518–20. doi: 10.1126/science.271.5248.518 8560269

[72] Dupont S, Morsut L, Aragona M, Enzo E, Giulitti S, Cordenonsi M, et al. Role of YAP/TAZ in mechanotransduction. Nature. 2011;474: 179–183. doi: 10.1038/nature10137 21654799

[73] Lampi MC, Reinhart-King CA. Targeting extracellular matrix stiffness to attenuate disease: From molecular mechanisms to clinical trials. Sci Transl Med. 2018;10: eaao0475. doi: 10.1126/scitranslmed.aao0475 29298864

[74] Fang Y, Wu D, Birukov KG. Mechanosensing and Mechanoregulation of Endothelial Cell Functions. 2019;9: 873–904. doi: 10.1002/cphy.c180020 30873580 PMC6697421

[75] Yoon J, Chung J, Hwa K, Hyun S, Kim M, Park J, et al. Fluid shear stress regulates the expression of Lectin-like oxidized low density lipoprotein receptor-1 via KLF2-AP-1 pathway depending on its intensity and pattern in endothelial cells. Atherosclerosis. 2018;270: 76–88. doi: 10.1016/j.atherosclerosis.2018.01.038 29407891

[76] Hermonat PL, Zhu H, Cao M. LOX-1 Transcription. 2011; 393–400. doi: 10.1007/s10557-011-6322-8 21796333

[77] Zhao W, Ma G, Chen X. Lipopolysaccharide induced LOX-1 expression via TLR4 / MyD88 / ROS activated p38MAPK-NF- κ B pathway. Vascul Pharmacol. 2014;63: 162–172. doi: 10.1016/j.vph.2014.06.008 25135647

[78] Bae YH, Mui KL, Hsu BY, Liu SL, Cretu A, Razinia Z, et al. A FAK-Cas-Rac-lamellipodin signaling module transduces extracellular matrix stiffness into mechanosensitive cell cycling. Sci Signal. 2014;7. doi: 10.1126/scisignal.2004838 24939893 PMC4345117

[79] Qin C, Nagao T, Grosheva I, Maxfield FR, Pierini LM. Elevated plasma membrane cholesterol content alters macrophage signaling and function. Arterioscler Thromb Vasc Biol. 2006;26. doi: 10.1161/01.ATV.0000197848.67999.e1 16306428

[80] Bandaru S, Ala C, Ekstrand M, Akula MK, Pedrelli M, Liu X, et al. Lack of RAC1 in macrophages protects against atherosclerosis. PLoS One. 2020;15. doi: 10.1371/journal.pone.0239284 32941503 PMC7498073

[81] Yusuf B, Mukovozov I, Patel S, Huang YW, Liu GY, Reddy EC, et al. The neurorepellent, Slit2, prevents macrophage lipid loading by inhibiting CD36-dependent binding and internalization of oxidized low-density lipoprotein. Sci Rep. 2021;11. doi: 10.1038/s41598-021-83046-x 33574432 PMC7878733

[82] Ding L, Zhang L, Kim M, Byzova T, Podrez E. Akt3 kinase suppresses pinocytosis of low-density lipoprotein by macrophages via a novel WNK/SGK1/Cdc42 protein pathway. J Biol Chem. 2017;292: 9283–9293. doi: 10.1074/jbc.M116.773739 28389565 PMC5454109

[83] Xu S, Liu B, Yin M, Koroleva M, Mastrangelo M, Ture S, et al. A novel TRPV4-specific agonist inhibits monocyte adhesion and atherosclerosis. Oncotarget. 2016;7. doi: 10.18632/oncotarget.9376 27191895 PMC5122337

[84] Józefowski S, Biedroń R, Sróttek M, Chadzińska M, Marcinkiewicz J. The class A scavenger receptor SR-A/CD204 and the class B scavenger receptor CD36 regulate immune functions of macrophages differently. Innate Immun. 2013/11/20. 2014;20: 826–847. doi: 10.1177/1753425913510960 24257313

[85] Orekhov AN, Oishi Y, Nikiforov NG, Zhelankin A V, Sobenin IA, Kel A, et al. Modified LDL Particles Activate Inflammatory Pathways in Monocyte-derived Macrophages: Transcriptome Analysis. 2018; 3143–3151. doi: 10.2174/1381612824666180911120039 30205792 PMC6302360

[86] Frisdal E, Lesnik P, Olivier M, Robillard P, Chapman MJ, Huby T, et al. Interleukin-6 Protects Human Macrophages from Cellular Cholesterol Accumulation and Attenuates the Proinflammatory Response. J Biol Chem. 2011;286: 30926–30936. doi: 10.1074/jbc.M111.264325 21757719 PMC3162452

[87] Bisgaard LS, Mogensen CK, Rosendahl A, Cucak H, Nielsen LB, Rasmussen SE, et al. Bone marrow-derived and peritoneal macrophages have different inflammatory response to oxLDL and M1/M2 marker expression—Implications for atherosclerosis research. Sci Rep. 2016;6: 1–10. 27734926 10.1038/srep35234 PMC5062347

[88] Moore KJ, Kunjathoor V V., Koehn SL, Manning JJ, Tseng AA, Silver JM, et al. Loss of receptor-mediated lipid uptake via scavenger receptor A or CD36 pathways does not ameliorate atherosclerosis in hyperlipidemic mice. J Clin Invest. 2005;115: 2192–2201. doi: 10.1172/JCI24061 16075060 PMC1180534

[89] Barczyk M, Carracedo S, Gullberg D. Integrins. Cell Tissue Res. 2010;339: 269–280. doi: 10.1007/s00441-009-0834-6 19693543 PMC2784866

[90] Hocking DC, Sottile J, McKeown-Longo PJ. Activation of Distinct α_5_ β_1_-mediated Signaling Pathways by Fibronectin’s Cell Adhesion and Matrix Assembly Domains. J Cell Biol. 1998;141: 241–253. doi: 10.1083/jcb.141.1.241 9531562 PMC2132721

[91] Takahashi S, Leiss M, Moser M, Ohashi T, Kitao T, Heckmann D, et al. The RGD motif in fibronectin is essential for development but dispensable for fibril assembly. J Cell Biol. 2007;178: 167–178. doi: 10.1083/jcb.200703021 17591922 PMC2064432

[92] Farias E, Lu M, Li X, Schnapp LM. Integrin α8β1–fibronectin interactions promote cell survival via PI3 kinase pathway. Biochem Biophys Res Commun. 2005;329: 305–311. doi: 10.1016/j.bbrc.2005.01.125 15721307

[93] Ahmed N, Riley C, Rice G, Quinn M. Role of Integrin Receptors for Fibronectin, Collagen and Laminin in the Regulation of Ovarian Carcinoma Functions in Response to a Matrix Microenvironment. Clin Exp Metastasis. 2005;22: 391–402. doi: 10.1007/s10585-005-1262-y 16283482

[94] Lacouture ME, Schaffer JL, Klickstein LB. A comparison of type I collagen, fibronectin, and vitronectin in supporting adhesion of mechanically strained osteoblasts. J Bone Miner Res. 2002;17: 481–92. doi: 10.1359/jbmr.2002.17.3.481 11874239

[95] Koyama Y, Norose-Toyoda K, Hirano S, Kobayashi M, Ebihara T, Someki I, et al. Type I collagen is a non-adhesive extracellular matrix for macrophages. Arch Histol Cytol. 2000;63: 71–9. doi: 10.1679/aohc.63.71 10770590

[96] Danen EHJ, Sonneveld P, Brakebusch C, Fässler R, Sonnenberg A. The fibronectin-binding integrins α5β1 and αvβ3 differentially modulate RhoA–GTP loading, organization of cell matrix adhesions, and fibronectin fibrillogenesis. J Cell Biol. 2002;159: 1071. doi: 10.1083/jcb.200205014 12486108 PMC2173988

[97] Huveneers S, Truong H, Fassler R, Sonnenberg A, Danen EHJ. Binding of soluble fibronectin to integrin 5 1—link to focal adhesion redistribution and contractile shape. J Cell Sci. 2008;121: 2452–2462. doi: 10.1242/jcs.033001 18611961

[98] Yurdagul A, Green J, Albert P, McInnis MC, Mazar AP, Orr AW. 5 1 Integrin Signaling Mediates Oxidized Low-Density Lipoprotein-Induced Inflammation and Early Atherosclerosis. Arterioscler Thromb Vasc Biol. 2014;34: 1362–1373. doi: 10.1161/ATVBAHA.114.303863 24833794 PMC4096780

[99] Seo JW, Yang EJ, Yoo KH, Choi IH. Macrophage differentiation from monocytes is influenced by the lipid oxidation degree of low density lipoprotein. Mediators Inflamm. 2015;2015. doi: 10.1155/2015/235797 26294848 PMC4532889

[100] Radhika A, Sudhakaran PR. Upregulation of macrophage-specific functions by oxidized LDL: Lysosomal degradation-dependent and -independent pathways. Mol Cell Biochem. 2013;372: 181–190. doi: 10.1007/s11010-012-1459-8 23054190

[101] Miller YI, Viriyakosol S, Binder CJ, Feramisco JR, Kirkland TN, Witztum JL. Minimally modified LDL binds to CD14, induces macrophage spreading via TLR4/MD-2, and inhibits phagocytosis of apoptotic cells. J Biol Chem. 2003;278: 1561–1568. doi: 10.1074/jbc.M209634200 12424240

[102] Kavanagh IC, Symes CE, Renaudin P, Nova E, Mesa MD, Boukouvalas G, et al. Degree of oxidation of low density lipoprotein affects expression of CD36 and PPARγ, but not cytokine production, by human monocyte-macrophages. Atherosclerosis. 2003;168: 271–282. doi: 10.1016/s0021-9150(03)00148-5 12801610

[103] Glass CK, Witztum JL. Atherosclerosis. the road ahead. Cell. 2001;104: 503–16. doi: 10.1016/s0092-8674(01)00238-0 11239408

[104] Nunes P, Demaurex N. The role of calcium signaling in phagocytosis. J Leukoc Biol. 2010;88. doi: 10.1189/jlb.0110028 20400677

[105] Getz GS, Reardon C a. Animal models of Atherosclerosis. Arterioscler Thromb Vasc Biol. 2012;32: 1104–1115. doi: 10.1161/ATVBAHA.111.237693 22383700 PMC3331926

[106] Chanput W, Mes JJ, Wichers HJ. THP-1 cell line: An in vitro cell model for immune modulation approach. International Immunopharmacology. 2014. doi: 10.1016/j.intimp.2014.08.002 25130606

[107] Pinto SM, Kim H, Subbannayya Y, Giambelluca MS, Bösl K, Ryan L, et al. Comparative Proteomic Analysis Reveals Varying Impact on Immune Responses in Phorbol 12-Myristate-13-Acetate-Mediated THP-1 Monocyte-to-Macrophage Differentiation. Front Immunol. 2021;12. doi: 10.3389/fimmu.2021.679458 34234780 PMC8255674

[108] Bosshart H, Heinzelmann M. THP-1 cells as a model for human monocytes. Annals of Translational Medicine. AME Publishing Company; 2016. doi: 10.21037/atm.2016.08.53 27942529 PMC5124613

[109] An D, Hao F, Zhang F, Kong W, Chun J, Xu X, et al. CD14 is a key mediator of both lysophosphatidic acid and lipopolysaccharide induction of foam cell formation. J Biol Chem. 2017;292. doi: 10.1074/jbc.M117.781807 28705936 PMC5582834

