# Supplementary figures and images for: Macrophage uptake of oxidized and acetylated low-density lipoproteins and generation of reactive oxygen species are regulated by linear stiffness of the growth surface
Source: PLoS One. 2021 Dec 16;16(12):e0260756. doi: 10.1371/journal.pone.0260756 (PMC8675690; doi:10.1371/journal.pone.0260756)

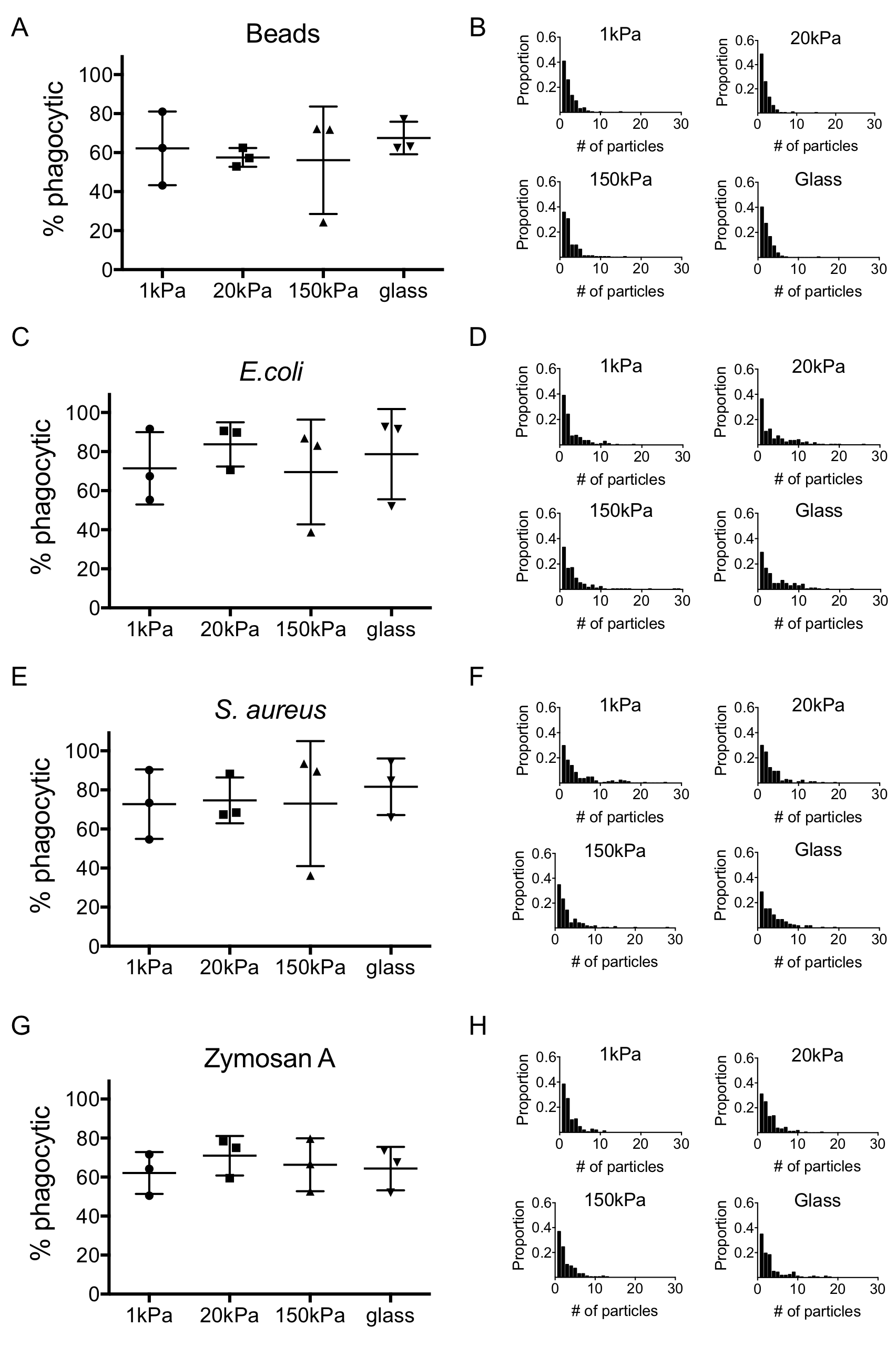

Supplement: S1 Fig — Primary bone marrow-derived macrophages BMMs were grown on fibronectin-coated 1, 20, 150 kilopascal (kPa) polyacrylamide gels or fibronectin-coated glass for 24h. BMMs were incubated with fluorescently-labeled (A-B) silica beads, (C-D) E.coli, (E-F) S.aureus, or (G-H) Zymosan A for 5min, fixed in 3% paraformaldehyde, stained with phalloidin (F-actin), and imaged by epifluorescence microscopy. (A, C, E, G) show the percentage of BMMs on each of the substrates that phagocytosed at least one particle. Mean +/- SD from three independent experiments is shown. Data were analyzed by one-way ANOVA with Tukey’s multiple comparisons test; no significant differences were reported. (B, D, F, H) The number of internalized particles per cell from a minimum of 50 BMMs per condition in three independent experiments were quantified. Results were analyzed by the Kruskal-Wallis test. (TIF) [file pone.0260756.s001.tif]

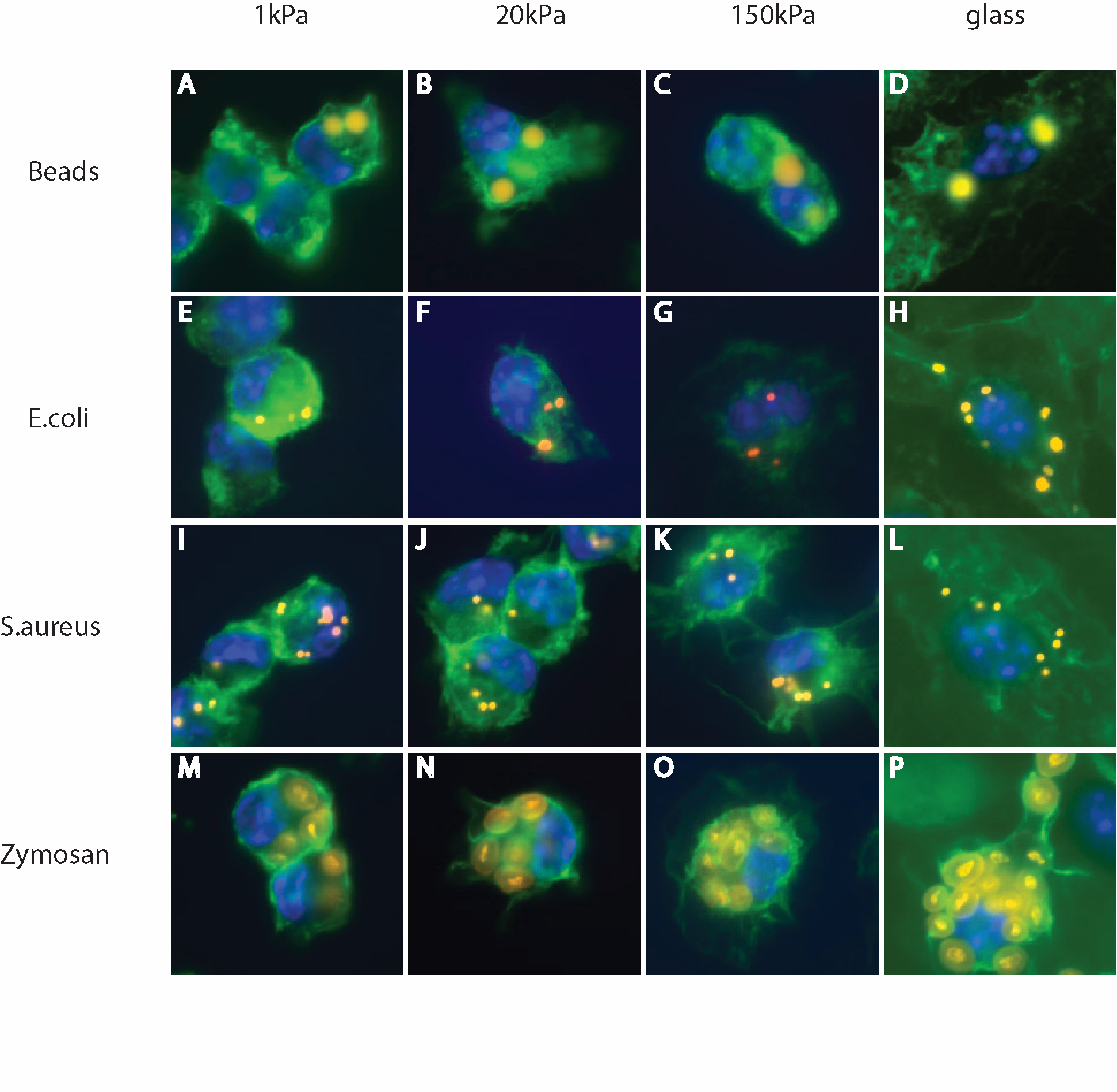

Supplement: S2 Fig — Primary bone marrow-derived macrophages BMMs were grown on fibronectin-coated 1, 20, 150 kilopascal (kPa) polyacrylamide gels or fibronectin-coated glass for 24h. BMMs were incubated with fluorescently-labeled (A-D) silica beads, (E-H) E.coli, (I-L) S.aureus, or (M-P) Zymosan A for 30min, fixed in 3% paraformaldehyde, stained with phalloidin (F-actin), and imaged with an Axio Imager M1 microscope. Original objective magnification 63x, scale bar = 10μm. (TIFF) [file pone.0260756.s002.tiff]

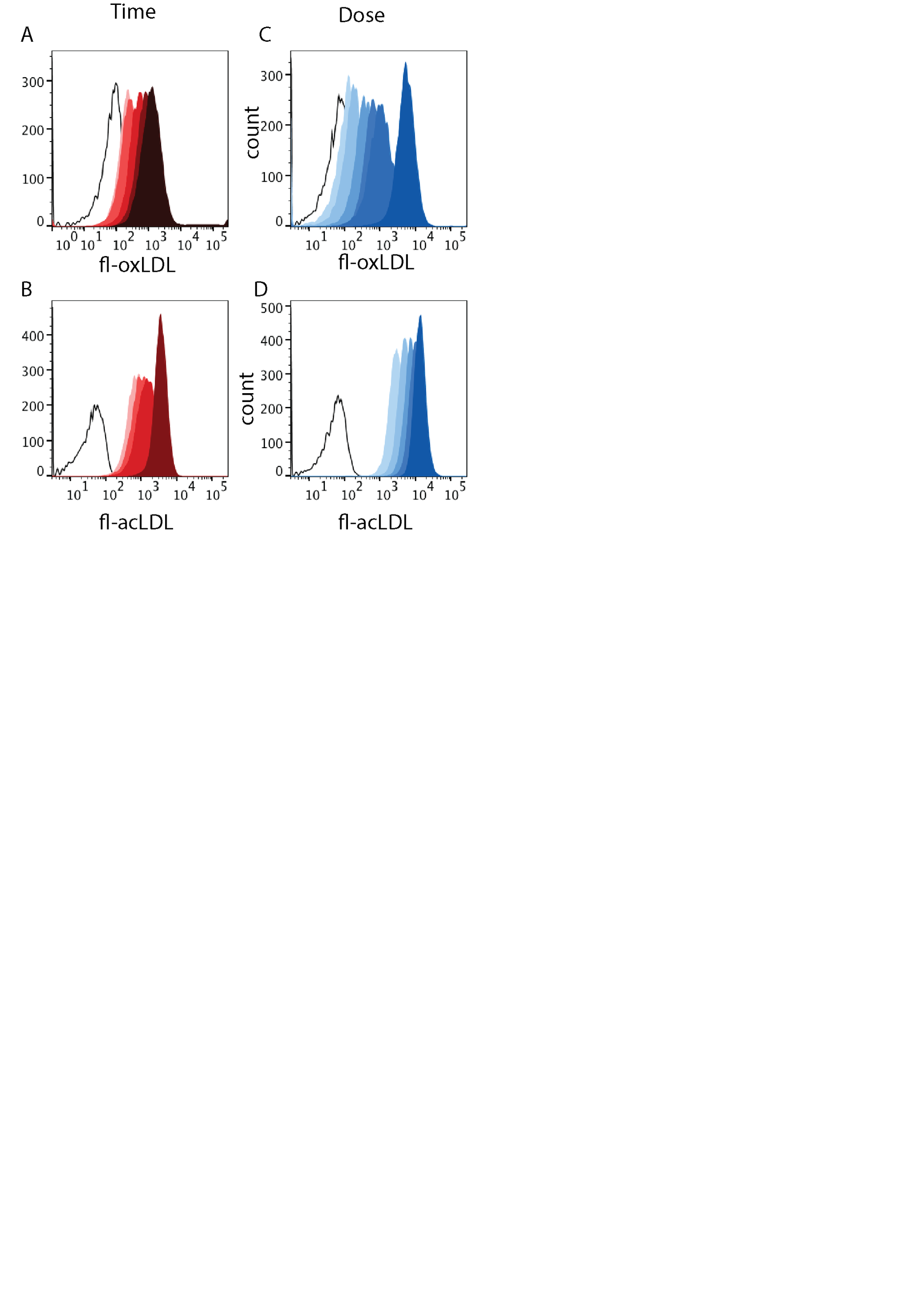

Supplement: S3 Fig — Primary bone marrow-derived macrophages (BMMs) were grown on fibronectin-coated glass for 24 hours, treated as indicated, and then removed into a single cell suspension for analysis by flow cytometry. Treatments were as follows: A) fluorescently labeled oxidized LDL (fl-oxLDL) at final concentrations of 0.5, 1, 2, 4, 8 μg/ml) for 4 hours; B) fluorescently labeled acetylated LDL (fl-acLDL) at final concentration of 0.5, 1, 2, 6, 10 μg/ml) for 4 hours. C) fl-oxLDL (2μg/ml) for 0.5, 1, 2, 4, 6, 24 hours; or D) fl-acLDL (2μg/ml) for 0.5, 1, 2, 4, 24 hours. Darker shades of red correspond to higher concentrations of fl-oxLDL (A) or fl-acLDL (B). Darker shades of blue correspond to longer incubation times with fl-oxLDL (C) or fl-acLDL (D). Data are representative of a minimum of two independent experiments. (TIF) [file pone.0260756.s003.tif]

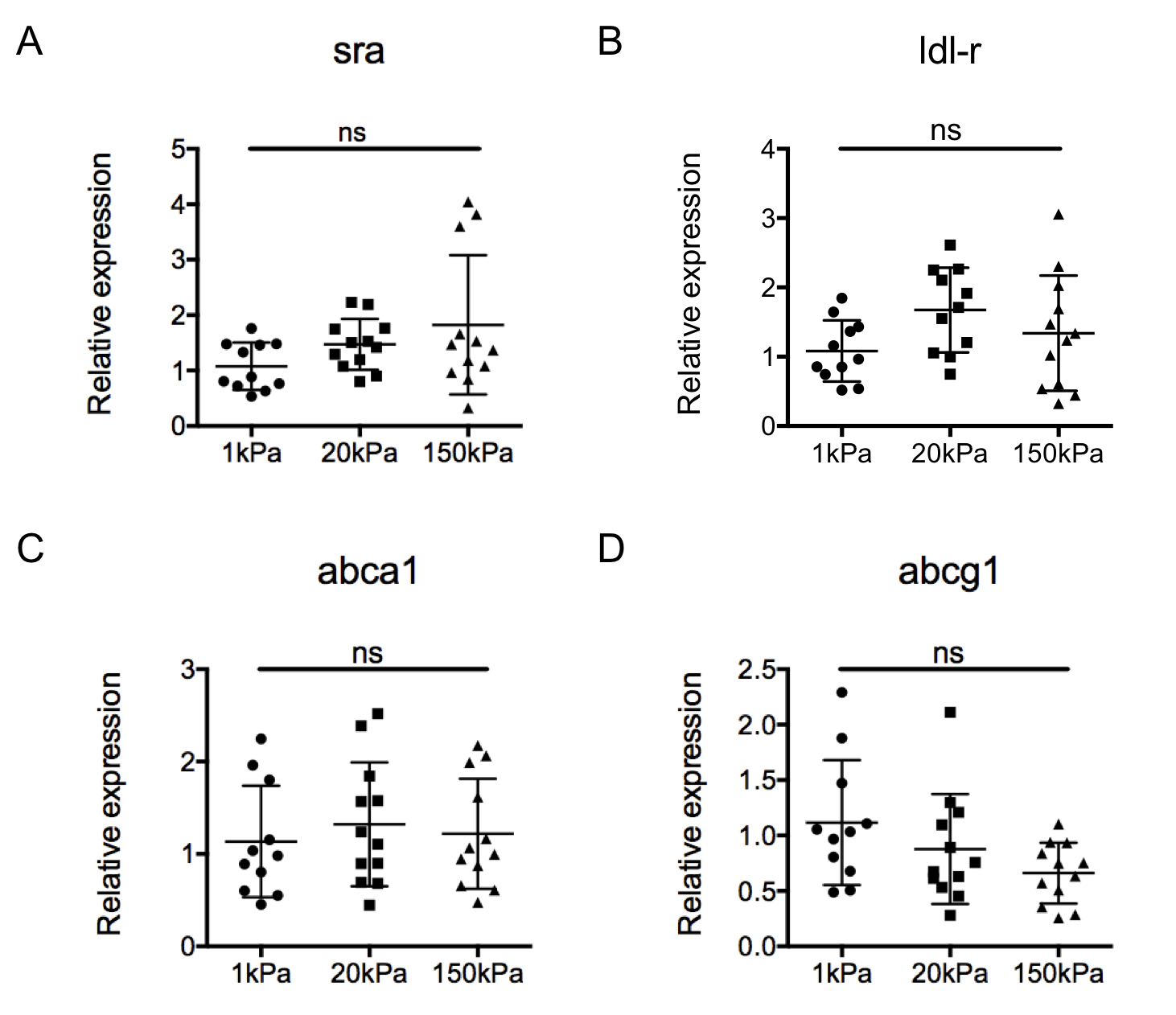

Supplement: S4 Fig — Primary bone marrow-derived macrophages (BMMs) were grown on fibronectin-coated 1, 20, 150 kilopascal (kPa) polyacrylamide gels for 24 hours. Relative gene expression was quantified by quantitative PCR using the ΔΔCt method and 18S for normalization. A) scavenger receptor A (sra); B) LDL receptor (ldl-r); C) ATP binding cassette transporter A1 (abca1); D) abcg1. Results were analyzed by one-way ANOVA with Tukey’s multiple comparisons test. (TIF) [file pone.0260756.s004.tif]

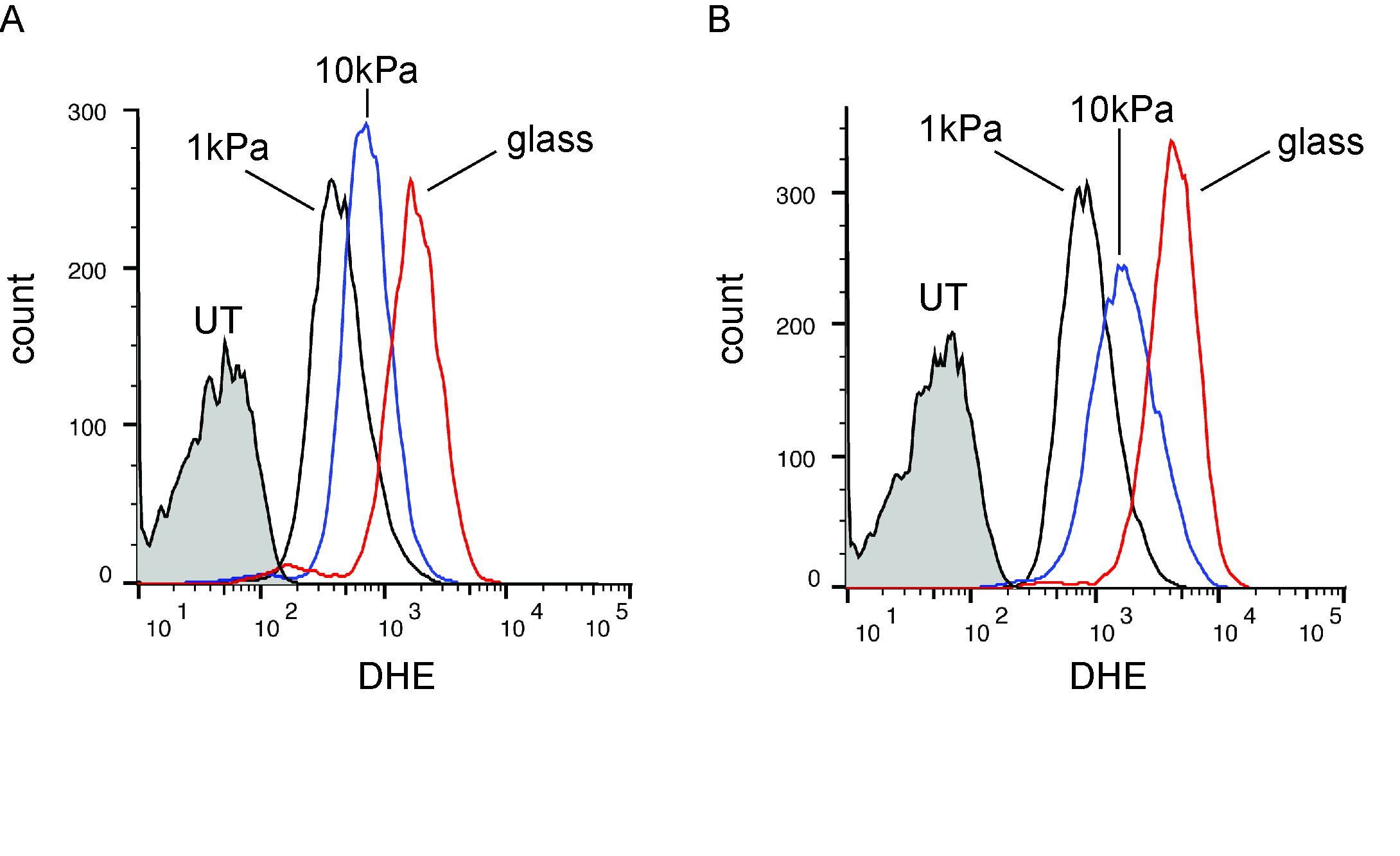

Supplement: S5 Fig — Primary bone marrow-derived macrophages (BMMs) were grown on fibronectin-coated 1 or 10 kilopascal (kPa) polyacrylamide gels or glass for 24 hours. Media was replaced with Hank’s buffered saline (UT, grey-filled) or dihydroethidium (DHE, 10μM) in Hank’s buffered saline for 45 minutes. Cells were washed and removed for analysis by flow cytometry. B) As in (A), except that BMMs were grown on each surface for 48 hours prior to treatment with and without DHE. Gray shaded = BMMs without DHE (UT); Open black line = BMMs on 1kPa incubated with DHE. Open blue line = BMMs on 10kPa incubated with DHE. Open red line = BMMs on glass incubated with DHE. Data are representative of a minimum of two independent experiments. (TIF) [file pone.0260756.s005.tif]
